# Intestinal *Apc*‐inactivation induces HSP25 dependency

**DOI:** 10.15252/emmm.202216194

**Published:** 2022-11-02

**Authors:** Sanne M van Neerven, Wouter L Smit, Milou S van Driel, Vaishali Kakkar, Nina E de Groot, Lisanne E Nijman, Clara C Elbers, Nicolas Léveillé, Jarom Heijmans, Louis Vermeulen

**Affiliations:** ^1^ Laboratory for Experimental Oncology and Radiobiology, Center for Experimental and Molecular Medicine Amsterdam UMC Location University of Amsterdam Amsterdam The Netherlands; ^2^ Cancer Center Amsterdam Amsterdam The Netherlands; ^3^ Amsterdam Gastroenterology Endocrinology Metabolism Amsterdam The Netherlands; ^4^ Oncode Institute Amsterdam The Netherlands; ^5^ Department of Gastroenterology and Hepatology, Tytgat Institute for Liver and Intestinal Research Amsterdam UMC Location University of Amsterdam Amsterdam The Netherlands; ^6^ Department of Internal Medicine Amsterdam UMC Location University of Amsterdam Amsterdam The Netherlands

**Keywords:** *Apc* mutations, colorectal cancer, heat shock proteins, intestinal stem cells, Wnt signaling, Cancer, Digestive System

## Abstract

The majority of colorectal cancers (CRCs) present with early mutations in tumor suppressor gene *APC*. *APC* mutations result in oncogenic activation of the Wnt pathway, which is associated with hyperproliferation, cytoskeletal remodeling, and a global increase in mRNA translation. To compensate for the increased biosynthetic demand, cancer cells critically depend on protein chaperones to maintain proteostasis, although their function in CRC remains largely unexplored. In order to investigate the role of molecular chaperones in driving CRC initiation, we captured the transcriptomic profiles of murine wild type and *Apc‐*mutant organoids during active transformation. We discovered a strong transcriptional upregulation of *Hspb1*, which encodes small heat shock protein 25 (HSP25). We reveal an indispensable role for HSP25 in facilitating *Apc*‐driven transformation, using both *in vitro* organoid cultures and mouse models, and demonstrate that chemical inhibition of HSP25 using brivudine reduces the development of premalignant adenomas. These findings uncover a hitherto unknown vulnerability in intestinal transformation that could be exploited for the development of chemopreventive strategies in high‐risk individuals.

## Graphical abstract

Upon loss of *Apc*, the most common early genetic lesion in colorectal cancer, cells upregulate a genetic program involved in buffering proteotoxic stress, including small heat shock protein HSP25. Here, we reveal that inhibition of HSP25 inhibits oncogenic transformation and prevents polyp formation.

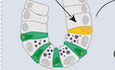

## Introduction

The development of colorectal cancer (CRC) is characterized by the stepwise accumulation of mutations (Fearon & Vogelstein, 1990). More than 80% of all CRCs present with early mutations in the tumor suppressor gene *APC*, leading to overactivation of the Wnt pathway and the formation of premalignant polyps (Muzny *et al*, 2012). *Apc*‐driven transformation of the intestinal epithelium is associated with hyperproliferation, impaired differentiation, and massive cytoskeletal remodeling (Sansom *et al*, 2004; Näthke, 2006; Barker *et al*, 2009). In order to fulfill the biosynthetic demands associated with these processes, *Apc*‐mutant cells increase their rate of global mRNA translation (Faller *et al*, 2014; Smit *et al*, 2020a) through a coordinated transcriptional network downstream of oncogenic c‐MYC and effector functions of mTORC1 signaling on mRNA translation (Van Riggelen *et al*, 2010). We have previously shown that proteotoxic stress, which is closely linked to increased protein synthesis levels, compromises the intestinal stem cell state and renders *Apc*‐mutant cells vulnerable to endoplasmic reticulum (ER) stress (van Lidth de Jeude *et al*, 2018; Meijer *et al*, 2021). Since the ER is mainly involved in posttranslational folding and processing of extracellular proteins, which are not directly used for the purpose of cell division or cytoskeletal reorganization, additional factors must be at play to ensure protein integrity in these hyperproliferative *Apc*‐mutant cells.

Heat shock proteins (HSPs) are a diverse class of molecular chaperones that perform multiple intracellular tasks related to the preservation of proteostasis in conditions of stress. In addition, HSPs are essential for cancer development due to their multifaceted role in controlling proliferation, differentiation, apoptosis, angiogenesis, and metastasis (Calderwood *et al*, 2006). It is becoming increasingly evident that HSPs also play a crucial role in controlling tumor initiation. For example, squamous cell carcinoma cells with oncogenic mutations in *KRAS* and *TP53* that lack *HSF1*, a master transcriptional regulator that activates the heat shock pathway, are refractory to oncogenic transformation (Dai *et al*, 2007). In addition, loss of *Hsp70* prevents the initiation of mammary tumors (Gong *et al*, 2015), indicating that oncogenic transformation itself may impose cellular proteotoxic stress and dependency on HSPs. Although the functions of protein chaperones in promoting cancer initiation and development are increasingly recognized, the influence of HSPs involved in *Apc*‐driven oncogenic transformation of intestinal epithelial cells is currently poorly defined.

In this study, we sought to identify molecular chaperones involved in *Apc*‐driven transformation by capturing the transcriptomic profiles of murine small intestinal organoids directly after homozygous deletion of *Apc*. We detected a significant upregulation of *Heat shock protein B1* (*Hspb1*), which encodes HSP25, a chaperone known to be involved in the refolding of proteins in conditions of cellular stress, the inhibition of apoptosis, and remodeling of the actin cytoskeleton (Arrigo, 2017). We confirmed exclusive expression of *Hspb1* in murine and human adenomas and revealed that upregulation of HSP25 is Wnt‐driven. Furthermore, we discovered that CRISPR/Cas9‐mediated knockout of *Hspb1* or chemical inhibition of HSP25 using the antiviral compound brivudine (Bromovinyldeoxyuridine, BVDU) drastically impaired oncogenic transformation *in vitro*. Furthermore, oral administration of BVDU in a conditional knockout mouse model for *Apc* significantly reduced the abundance and expansion of *Apc*‐mutant crypts. In line with this, short‐term administration of BVDU resulted in a decrease in adenoma formation and increased survival rates. Taken together, our results indicate that *Apc*‐mutant cells are critically dependent on HSP25, and inhibition of this small heat shock protein poses a new therapeutic target for the prevention of premalignant adenomas.

## Results

### Apc‐driven intestinal transformation results in increased expression of Hspb1

**Figure 1:**
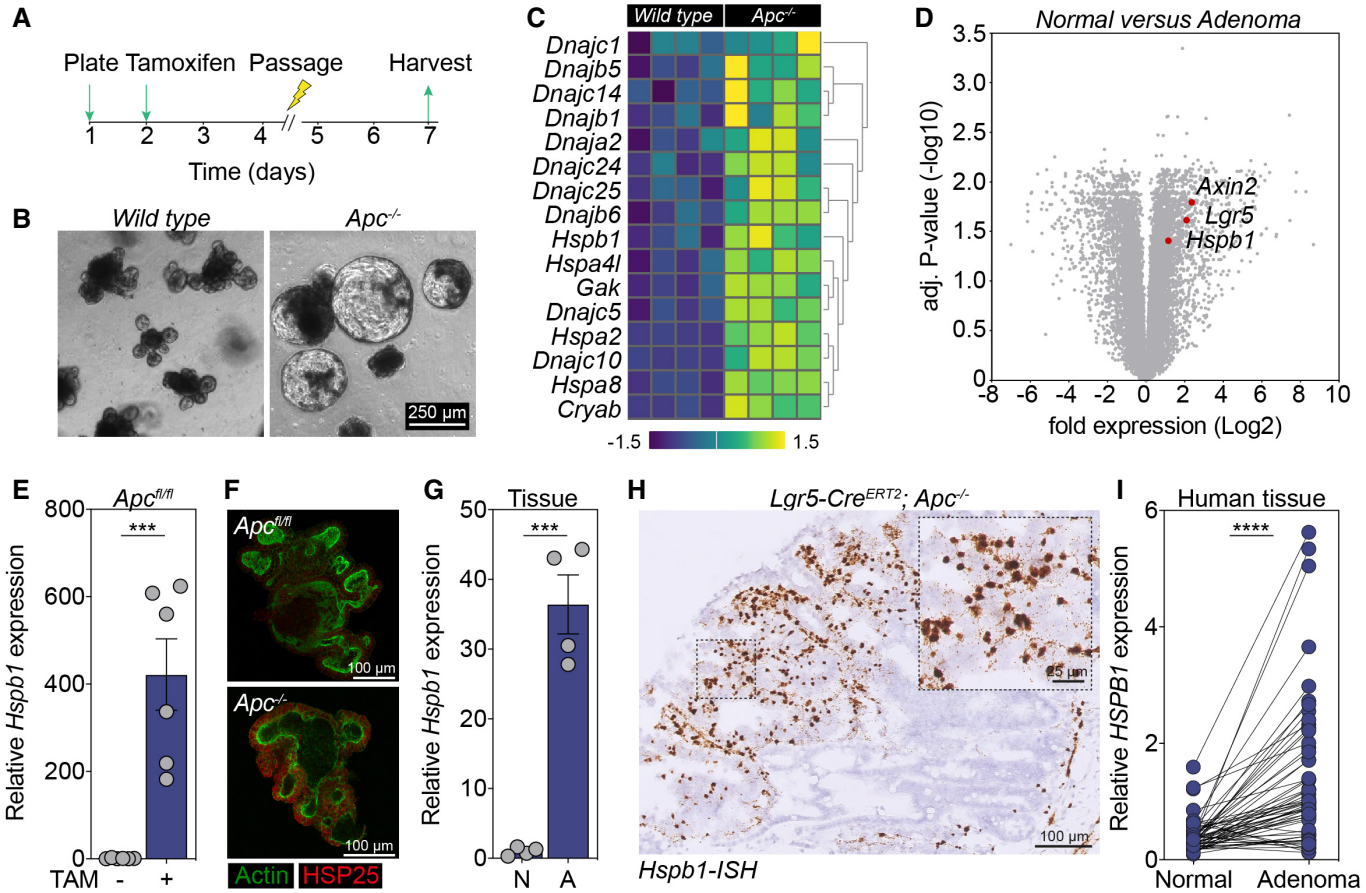
Identification of *Hspb1* as *Apc*‐specific heat shock protein ASchematic illustration of the *in vitro* recombination experiment. TAM, 4OH‐tamoxifen.BRepresentative phase images of the morphology of WT and *Apc* ^−/−^ organoids before mRNA isolation. Scalebar, 250 μm.CHeat map illustrating differentially expressed genes (DEGs) encoding heat shock proteins between WT and *Apc* ^−/−^ organoids.DVolcano plot of DEGs between normal mouse intestinal tissue and adenomas (GSE65461).E, FRelative gene expression of *Hspb1* (***P = 0.0004, *n* = 6) (E) and protein expression of HSP25 (F) in unrecombined versus recombined murine organoids.GGene expression of *Hspb1* in normal (N) versus adenoma (A) tissue (***P = 0.0002, *n* = 4).HRNA‐ISH for *Hspb1* in murine intestinal adenoma. Scalebar, 100 μm. Zoom panel, 25 μm.IRelative gene expression of human *HSPB1* in paired sets of normal versus adenoma tissue, (****P < 0.0001, paired *t*‐test, *n* = 59 pairs). Data information: All data are mean ± s.e.m., biological replicates, analyzed using unpaired two‐sided *t*‐test unless otherwise specified. Source data are available online for this figure.

**Figure EV1:**
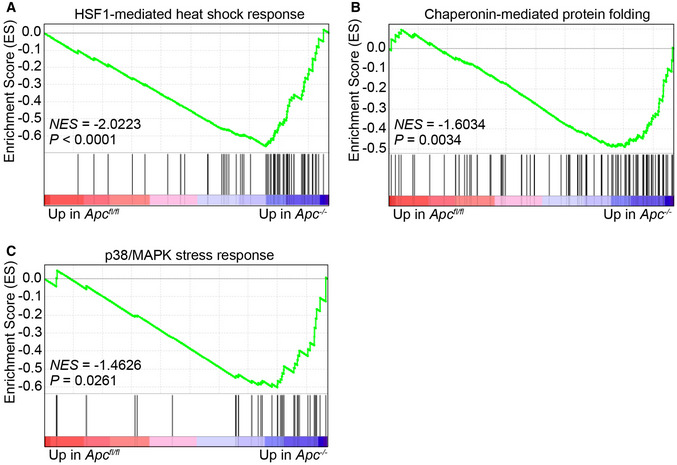
Loss of Apc induces cellular stress A–CGene Set Enrichment Analysis for pathways involved in HSF1‐mediated heat shock response (A), chaperonin‐mediated protein folding (B), and p38/MAPK stress response (C). NES, Normalized Enrichment Score; *P*, nominal *P*‐value of the enrichment score, which is based on a phenotype‐based permutation test procedure as described in more detail in (Subramanian *et al*, 2005). Source data are available online for this figure.

To identify genes that are upregulated in response to acute loss of *Apc*, we generated *in vitro* organoid cultures derived from *Villin‐Cre* ^*ERT2*^ (wild type, WT) and *Villin‐Cre* ^*ERT2*^;*Apc* ^*fl*/*fl*^ mice. *Apc* was conditionally inactivated by administration of 2 μM 4OH‐tamoxifen, and organoids were dissociated and repassaged to ensure full recombination (Fig 1A; Smit *et al*, 2020a, Data ref: Smit *et al*, 2020b). After passaging, *Apc*‐mutant organoids demonstrated cystic‐like growth, which is a typical sign of elevated Wnt‐β‐catenin signaling in intestinal organoids, while WT cultures maintained their crypt‐like phenotype (Fig 1B). Three days after reseeding, organoids were harvested and RNA was extracted for global gene expression analysis. We identified a set of genes belonging to the heat shock protein family that were specifically upregulated after *Apc*‐driven oncogenic transformation (Fig 1C), and enrichment in pathways indicative of proteotoxic and cellular stress (Fig EV1A–C). Of particular interest was *Hspb1*, which was the only gene that was also significantly upregulated in murine adenomas when compared with normal intestinal tissue, together with Wnt target genes *Axin2* and *Lgr5* (Fig 1D; Reed *et al*, 2015a, Data ref: Reed *et al*, 2015b). *Hspb1* encodes HSP25, which is an ATP‐independent chaperone that keeps its client protein‐folding intermediates in a folding‐competent state for the HSP70 chaperone machinery during conditions of stress (Ehrnsperger *et al*, 1997). Moreover, HSP25 also possesses extra‐chaperone functions, including cytoskeleton stabilization and antioxidant activity, making it a potentially relevant factor for adaptation to *Apc*‐driven transformation (Bakthisaran *et al*, 2015). We validated the specific expression of *Hsbp1* and the concomitant upregulation of HSP25 protein in *Apc* ^fl/fl^ organoids after *in vitro* recombination (Fig 1 E and F). Moreover, we detected and elevated *Hspb1* expression in intestinal adenomas collected from *Lgr5‐Cre* ^*ERT2*^;*Apc* ^*fl*/*fl*^ mice (Fig 1G) and spatially confirmed the expression of *Hspb1* to adenomatous regions by RNA *in situ* hybridization (RNA‐ISH; Fig 1H). Importantly, we confirmed upregulation of the human homolog *HSPB1* in adenoma tissues obtained from a cohort of 59 patients of which we received both adenomatous and adjacent intestinal tissue of normal macroscopic appearance, which suggests that *HSPB1* upregulation in *APC*‐mutant cells is conserved from mouse to human (Fig 1I).

**Figure 2:**
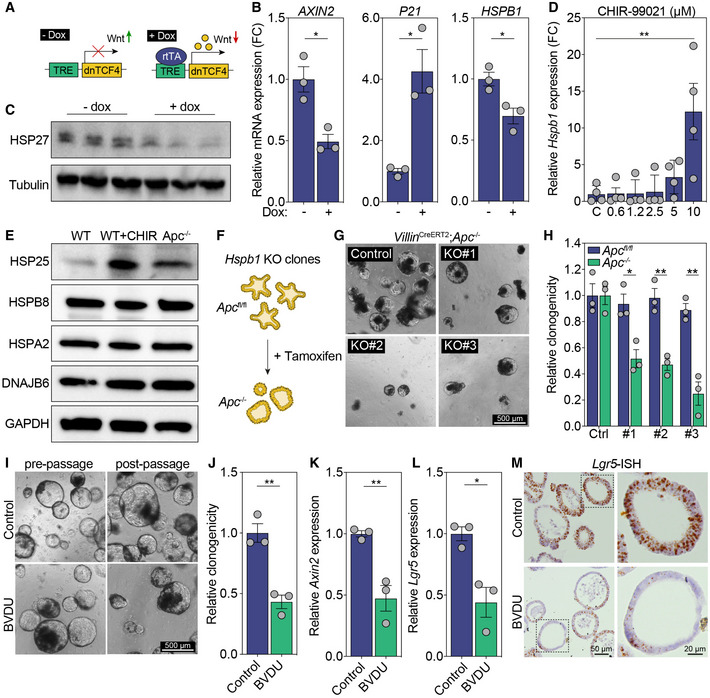
*Hspb1* expression is Wnt‐regulated and sensitizes *Apc*‐mutant cells to HSP25 inhibition *in vitro* ASchematic illustration of the doxycycline‐inducible dnTCF construct.B, CRelative gene expression of *AXIN2* (*P = 0.0129), *P21* (*P = 0.0103), and *HSPB1*(*P = 0.0236) (B), and protein expression of HSP27 (C) in Ls174t‐dnTCF4 cells in the presence or absence of doxycycline.D, ERelative gene expression of *Hspb1* in murine WT organoids treated with different concentrations of CHIR‐99021 (**P = 0.0016, one way ANOVA, *n* = 4) (D), and protein expression of HSP25 in WT and *Apc* ^−/−^ organoids (E).FSchematic illustration of *in vitro* recombination experiment using *Hspb1* KO clones.G, HRepresentative images of single cell *Hspb1* KO clones passaged after loss of *Apc* (G), and quantification of their clonogenic potential (*P = 0.0147(#1), **P = 0.0035(#2), **P = 0.0032(#3)) (H). Scale bar, 500 μm.I, JRepresentative images of *Apc* ^−/−^ organoids cultured in the absence or presence of 60 μM BVDU (I), and quantification of their clonogenic potential (**P = 0.0039) (J). Scale bar, 500 μm.K, LRelative gene expression of Wnt target genes *Axin2* (**P = 0.0080) and *Lgr5* (*P = 0.0142) in *Apc* ^−/−^ organoids cultured in the absence or presence of 60 μM BVDU.MRNA‐ISH for *Lgr5* in control or BVDU‐treated *Apc* ^−/−^ organoids. Scale bar, 50 μm, zoom panel 20 μm. Data information: All data are mean ± s.e.m. *n* = 3 biological replicates, analyzed using unpaired two‐sided *t*‐test unless otherwise specified. Source data are available online for this figure.

### Hspb1 is upregulated in response to Wnt pathway activation

To assess whether the upregulation of *Hspb1* is a direct result of Wnt/β‐catenin pathway activation, we used a β‐catenin mutant cancer cell line carrying a construct containing an inducible dominant negative TCF4 (dnTCF4), which upon doxycycline administration acts as inhibitor of the Wnt pathway (Fig 2A; Van de Wetering *et al*, 2002). As previously reported, incubation with doxycycline for 48 h revealed a marked decrease in Wnt target gene *AXIN2* and an increase in *P21* expression (Fig 2B; Van de Wetering *et al*, 2002). In addition, doxycycline treatment resulted in decreased *HSPB1* mRNA levels (Fig 2B) and downregulated HSP27 protein, the human ortholog of HSP25 (Fig 2C), indicating that *HSPB1* expression is TCF4‐dependent. Vice versa, activation of Wnt signaling by GSK3β inhibitor CHIR99021 increased *Hspb1* expression in a dose‐dependent manner in WT murine organoids (Fig 2D). In agreement, treatment with CHIR99021 increased HSP25 protein expression of WT organoids to comparable levels as *Apc*‐mutant organoids, while levels of other heat shock proteins remained unaffected (Fig 2E). Together, these data indicate that loss of *Apc* and the subsequent activation of Wnt signaling directly drive the upregulation of *Hspb1*.

### Inhibition and loss of HSP25 impairs oncogenic transformation *in vitro*

**Figure EV2:**
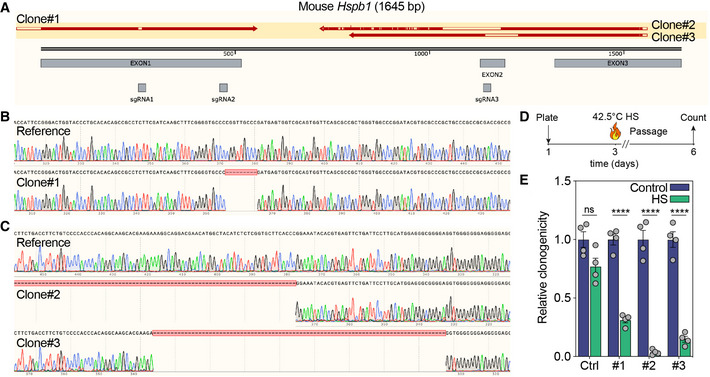
Validation of Hspb1 KO clones AOverview of Hspb1 gene, positions of the sgRNA's and edited sites within the KO clones.B, CSanger sequencing results of the edited regions in exon 1 (B) or exon 2 (C).D, EIllustration of the heat shock (HS) clonogenicity experiment (D), and quantification of clonogenicity in KO clones in untreated or HS‐treated conditions (****P < 0.0001 for KO#1, 2, and 3, *n* = 4 experiments, unpaired two‐sided *t*‐test). Source data are available online for this figure.

**Figure EV3:**
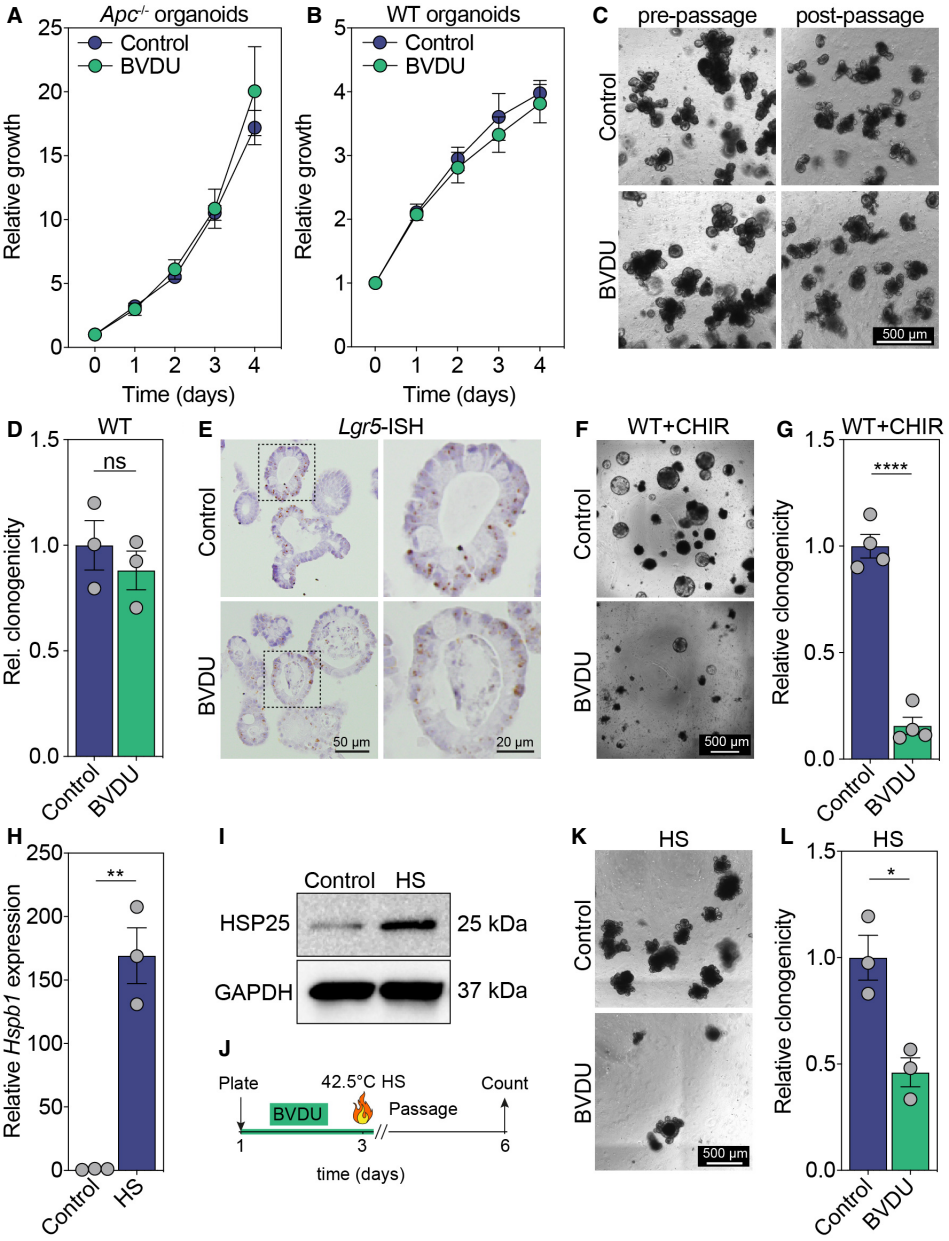
Effect of *in vitro* HSP25 inhibition using BVDU AGrowth curves of Apc‐mutant organoids.B–DGrowth curves of WT organoids (B), representative images of WT organoids cultured in the absence or presence of 60 μM BVDU (C), and quantification of their clonogenic potential (D). Scale bar, 500 μm.ERNA‐ISH for Lgr5 in control or BVDU‐treated WT organoids. Scale bar, 50 μm, zoom panel 20 μm.F, GRepresentative images (F) and clonogenicity (G) of WT organoids treated with CHIR99021 in the absence or presence of BVDU. (****P < 0.0001, *n* = 4). Scale bar, 500 μm.H, IRelative Hspb1 (H) and HSP25 (I) expression in wild‐type organoids after heat shock (HS) treatment (**P = 0.0016).J–LIllustration of the heat shock (HS) clonogenicity experiment (J), representative images of control or BVDU‐treated WT organoids after HS treatment (K), and quantification of clonogenicity (*P = 0.0128) (L). Data information: All data are mean ± s.e.m., *n* = 3 biological replicates, analyzed using unpaired two‐sided *t*‐test. Source data are available online for this figure.

**Figure EV4:**
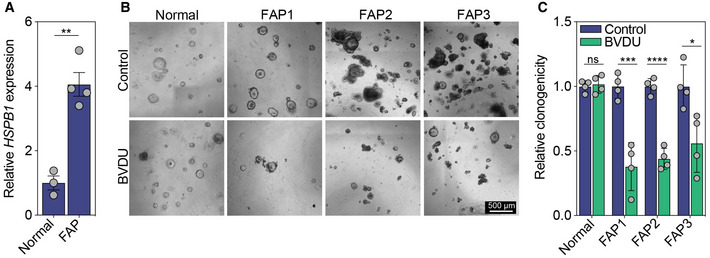
Effect of BVDU on human FAP organoids ARelative HSPB1 expression in normal colon organoids (*n* = 3) and organoids derived from patients with familial adenomatous polyposis (**P = 0.0013, *n* = 4, mean ± s.e.m.).B, Crepresentative images of control or BVDU‐treated human organoids (B) and quantification of clonogenicity (C) (***P = 0.0009 (FAP1), ****P < 0.0001 (FAP2), *P = 0.0197 (FAP3), *n* = 4 wells per line, data are mean ± s.d.) scale bar, 500 μm. Data information: All data are analyzed using unpaired two‐sided *t*‐test. Source data are available online for this figure.

Given the marked upregulation of *Hspb1* after *Apc*‐inactivation, we next assessed how loss of *Hspb1* influences oncogenic transformation using organoid cultures. We first generated single‐cell *Hspb1* knockout clones in a *Villin‐Cre* ^*ERT2*^;*Apc* ^*fl*/*fl*^ background using CRISPR/Cas9 (Fig EV2A–C) and administered tamoxifen to recombine *Apc in vitro* (Fig 2F). We observed that inactivation of *Hspb1* did not influence passaging potential of unrecombined (*Apc* ^*fl*/*fl*^) organoids (Fig 2G and H); however, conditional loss of *Apc* after tamoxifen administration significantly decreased outgrowth of transforming *Apc*‐mutant (*Apc* ^−/−^) organoids (Fig 2G and H). Moreover, chemical inhibition of HSP25 using BVDU demonstrated a comparable decrease in clonogenicity in established *Apc* ^−/−^ organoids (Fig 2I and J), which was accompanied by a decrease in expression of Wnt target genes *Axin2* and *Lgr5* (Fig 2K–M), while leaving growth of the organoids unaffected (Fig EV3A). Importantly, BVDU treatment did not influence growth, clonogenicity, or *Lgr5* expression in WT organoids (Fig EV3B–E), however, administration of both CHIR99021 and BVDU in WT organoids did result in loss of clonogenicity (Fig EV3F and G), confirming that HSP25 expression is Wnt regulated. Although the role of BVDU as HSP27 inhibitor has been described before (Heinrich *et al*, 2011), we confirmed functional inhibition of HSP25 by assessing recovery after heat shock stress, since protection against elevated temperatures is one of the best known roles of heat shock proteins (Landry *et al*, 1989). Short‐term heat shock of WT organoids resulted in a rapid increase in both *Hspb1* and HSP25 expression (Fig EV3H and I), and incubation with BVDU decreased clonogenic capacity after heat shock (Fig EV3J–L). Importantly, also the unrecombined (*Apc* ^fl/fl^) *Hspb1* KO clones were sensitive to heat shock, thereby functionally validating these clones (Fig EV2D and E). Together, these data reveal that *Apc*‐mutant epithelial cells are specifically sensitive to loss of *Hspb1* or inhibition of HSP25 during oncogenic transformation *in vitro*. We also observe increased *HSPB1* expression in human colon organoids derived from patients with *familial adenomatous polyposis* (FAP), carrying heritable mutations in the *APC* gene predisposing them to the development of adenomas and CRC (Fig EV4A) and confirmed that BVDU treatment decreased clonogenicity of these human FAP (*APC* ^−/−^) organoids (Fig EV4B and C).

**Figure 3:**
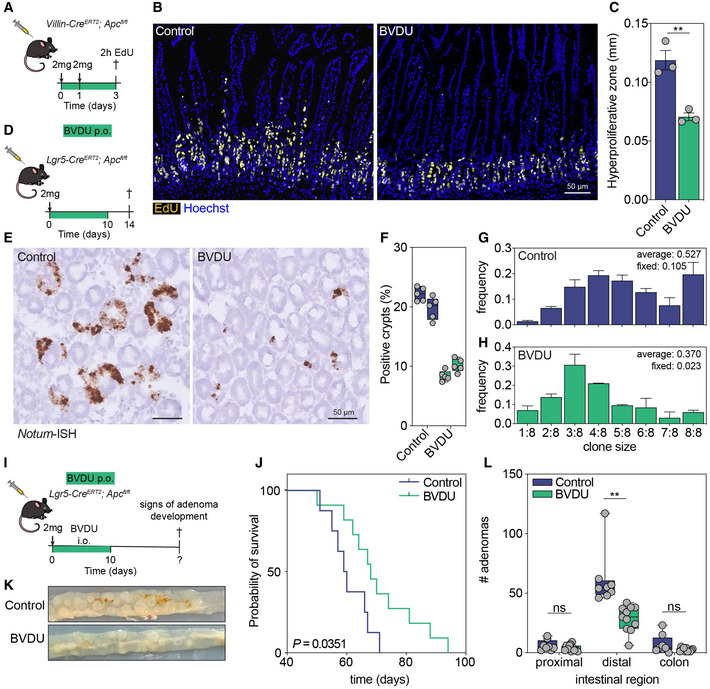
Inhibition of HSP25 prevents oncogenic transformation and adenoma development *in vivo* AExperimental set‐up of short‐term assay using *Villin‐Cre* ^*ERT2*^;*Apc* ^*fl*/*fl*^ mice. p.o., *per os*, orally.B, CVisualization of proliferating cells in tissues incubated with EdU for 2‐h (B), and quantification of the hyperproliferative zone (**P = 0.0057, *n* = 3 mice) (C). Scalebar, 50 μm.DExperimental set‐up of short‐term assay using *Lgr5‐Cre* ^*ERT2*^;*Apc* ^*fl*/*fl*^ mice.E, FRNA‐ISH for *Notum* reveals *Apc*‐mutant clones in crypt bottoms of control or BVDU‐treated mice (E), and quantification of the abundance of mutant crypts (*n* = 2 mice per condition, *n* = 5 technical replicates per mouse) (F). Scalebar, 50 μm.G, HClone size distributions of *Notum +* crypts in control (*n* = 265 crypts) or BVDU‐treated (*n* = 260 crypts) mice, the average clone size and number of fixed clones are included in the figure.IExperimental set‐up of tumor development assay using *Lgr5‐Cre* ^*ERT2*^;*Apc* ^*fl*/*fl*^ mice, mice were sacrificed once they develop signs of adenoma development.J–LSurvival curves for control and BVDU‐treated mice (*P* = 0.0351, Mantel‐Cox test) (J), macroscopic images of adenoma development in the distal small intestine (K), and boxplots for the number of adenomas per intestinal region (**P = 0.0013) (L), *n* = 8 control, *n* = 11 treated. Data information: All data are mean ± s.e.m., boxplots, the box represents the 25^th^–75^th^ percentile and the median (line), whiskers represent min to max values, biological replicates, analyzed using unpaired two‐sided *t*‐test unless otherwise specified. Source data are available online for this figure.

### Inhibition of HSP25 prevents oncogenic clonal expansion and adenoma formation *in vivo*

**Figure EV5:**
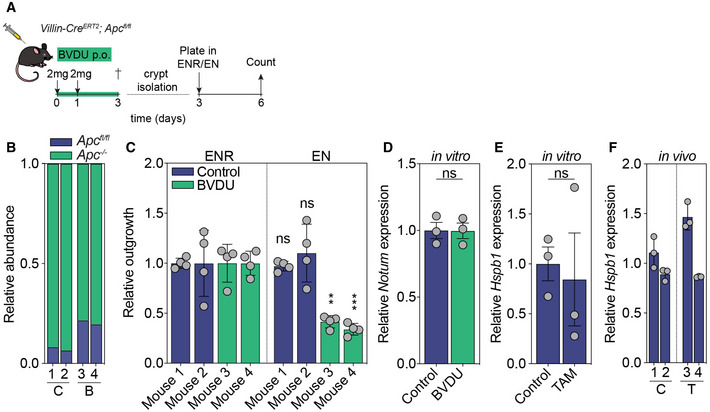
Validation of *in vivo* BVDU treatment AIllustration of experimental setup for crypt isolation.BRatio of unrecombined versus recombined Apc alleles in control and BVDU treated crypts (*n* = 2 mice per group, C, control, B, BVDU treated).COutgrowth of crypts isolated from control and BVDU‐treated mice in ENR (mEGF, Noggin, and Rspondin1) or EN medium (mEGF, Noggin) (**P = 0.0010 (mouse 3), ***P < 0.0001 (mouse 4), *n* = 4 wells per condition, data are mean ± s.d.).DRelative Notum expression in control or BVDU‐treated Apc organoids (*n* = 3 experiments).E, FRelative Hspb1 expression in control or tamoxifen‐treated organoids (*n* = 3 experiments) (E) or intestinal tissues (F) (*n* = 2 mice per condition, *n* = 3 technical replicates, C, control, T, Tamoxifen). Data information: All data are mean ± s.e.m., unless otherwise specified, analyzed using unpaired two‐sided *t*‐test. Source data are available online for this figure.

To study the effect of BVDU on *Apc*‐driven oncogenic transformation *in vivo*, we first used *Villin‐Cre* ^*ERT2*^;*Apc* ^*fl*/*fl*^ mice to activate epithelial‐wide recombination of *Apc* alleles in the intestine. As previously reported, upon injection of tamoxifen, these mice develop massive hyperproliferation throughout the epithelium followed by rapid death within a week (Heino *et al*, 2021). We injected two doses of 2 mg tamoxifen on two consecutive days, either in the presence or absence of BVDU (400 mg/kg), which was administered daily via oral gavage. Two hours before mice were sacrificed, they were injected with EdU to label actively dividing cells (Fig 3A). Visualization of EdU+ cells in intestinal tissues revealed a marked decrease in the length of the proliferative zone (Fig 3B and C), suggesting that BVDU treatment reversed the hyperproliferative phenotype associated with *Apc* loss. In line with these findings, isolation of crypts from control and BVDU‐treated mice revealed fewer recombined (*Apc* ^−/−^) alleles in BVDU exposed crypt fractions (Fig EV5A and B), as well as reduced outgrowth in selection medium that lacks R‐spondin1 (EN‐medium; Fig EV5C), both indicative of inhibition of oncogenic transformation. In order to assess the effect of BVDU on adenoma formation, we used *Lgr5‐Cre* ^*ERT2*^;*Apc* ^*fl*/*fl*^ mice, that allow for low‐dose recombination of *Apc* in the stem cell compartment and therefore is a more controlled model of intestinal transformation. First, we studied the short‐term effects of BVDU treatment on the number of mutant crypts (Fig 3D), which could be visualized by performing RNA *in situ* hybridization of *Notum*, a Wnt antagonist that was previously identified to be exclusively expressed by *Apc* ^−/−^ cells (Kleeman *et al*, 2020; Flanagan *et al*, 2021; van Neerven *et al*, 2021). Importantly, BVDU treatment did not influence *Notum* expression levels (Fig EV5D), nor did tamoxifen injection induce *Hspb1* expression (Fig EV5E and F). We demonstrated that daily treatment of BVDU for 10 days decreased the abundance of *Notum* ^*+*^ crypts (Fig 3E and F). In addition, we observed a reduction in clone sizes and the number of fully populated “fixed” *Notum* ^*+*^ crypts within single crypt bottoms 14 days after tamoxifen injection (Fig 3G and H). These data indicate that inhibition of HSP25 by using BVDU reduces expansion of *Apc*‐mutant clones and thus also potentially inhibits tumor initiation. Therefore, we performed a subsequent study in which we activated oncogenic Wnt signaling in *Lgr5‐Cre* ^*ERT2*^;*Apc* ^*fl*/*fl*^ mice in the absence or presence of BVDU for only 10 days surrounding the *Apc*‐inactivation and studied when these mice developed symptoms of adenoma development and needed to be sacrificed (Fig 3I). This revealed that BVDU‐treated mice display a significant survival advantage compared with untreated mice (Fig 3J). Of note, most BVDU mice eventually have to be sacrificed due to the development of a single tumor in the caecum that obstructed passage to the colon. Furthermore, assessment of the intestines of control and BVDU‐treated mice reveals a marked decrease in the number of adenomas, in particular in the distal region of the small intestine (Fig 3K and L). Taken together, our data reveal that *Apc*‐driven intestinal transformation drives the upregulation of *Hspb1* mRNA and HSP25 protein in a Wnt‐dependent fashion, and inhibition of HSP25 using BVDU only during the tumor initiation phase effectively reduces the subsequent development of premalignant adenomas.

## Discussion

In this study, we identified HSP25 as a critical mediator of oncogenic transformation following loss of tumor suppressor *Apc*. HSP25, a small heat shock protein with critical functions in regulating various stress responses, is upregulated after loss of *Apc* in organoid cultures and in murine adenomatous tissues in a Wnt‐dependent fashion. We demonstrate that *Apc*‐mutant organoids are particularly sensitive to loss of *Hspb1* or inhibition of HSP25 using BVDU, while growth kinetics and clonogenicity of WT organoids remain unaffected. Furthermore, BVDU administration significantly prevented oncogenic transformation and clonal expansion *in vivo*. Crucially, inhibition of HSP25 delayed the development and abundance of premalignant adenomas *in vivo*. Our study adds to the pleiotropic role of small HSPs such as HSP25 in facilitating CRC development, such as anti‐apoptotic effects, resistance to chemotherapeutics, and oncogenic mesenchymal signaling (Garrido *et al*, 1997; Henriques *et al*, 2018; Liu *et al*, 2020). However, in contrast to these studies, our data suggest that HSP25 is already crucial at an earlier stage by facilitating oncogenic transformation. This poses HSP25 inhibition using compounds such as BVDU as an interesting strategy to prevent tumor initiation, which is of particular relevance to high‐risk individuals, e.g., FAP patients. Importantly, clinical studies on the effects of BVDU for treatment of viral infections have reported minimal side effects in adults or children indicating potentially high tolerance to these agents (Wildiers & De Clercq, 1984; Benoit *et al*, 1985; Wassilew, 2005; Salvaggio & Gnann, 2017).

## Materials and Methods

### Animal experiments

This study made use of *Lgr5‐EGFP‐Cre* ^*ERT2*^, *Villin‐Cre* ^*ERT2*^, and *Apc* ^*fl*/*fl*^ mice that all have been described previously (Shibata *et al*, 1997; El Marjou *et al*, 2004; Barker *et al*, 2007) and were bred on a C57BL/6 background in the mouse facility of the Amsterdam UMC. All *in vivo* experiments are approved by the animal experimentation committee of the Amsterdam UMC – location AMC and are performed according to national guidelines under license number AVD1180020172125. Mice were housed under standard conditions, with temperatures between 20 and 24°C in 12‐h light/dark cycles with 40–70% humidity. For all studies, mice were between 6 and 12 weeks old at the start of the experiments. For short‐term experiments, both male and female mice were used, and for long‐term studies, only females were used. To assess the short‐term effect of brivudine (BVDU, MedChemExpress) on hyperproliferation upon conditional *Apc* loss, *Villin‐Cre* ^*ERT2*^;*Apc* ^*fl*/*fl*^ mice were injected intraperitoneally (i.p.) with 2 mg tamoxifen (Sigma) dissolved in cornflower oil for two consecutive days. Mice were randomly assigned into the control group or were treated with BVDU (400 mg/kg), which was administered daily via oral gavage (p.o.) during the entire duration of the experiment. In order to assess hyperproliferation, EdU (Thermo Fisher) was injected i.p. (100 μl of 10 mM stock) 2 h before mice were sacrificed. To study the effects of BVDU on stem cell dynamics and adenoma formation, *Lgr5‐EGFP‐Cre* ^*ERT2*^;*Apc* ^*fl*/*fl*^ mice were injected i.p. with a single dose of tamoxifen (2 mg) and randomly assigned to the control group or treated daily with BVDU for 10 consecutive days. For short‐term effects, mice were sacrificed 14 days after tamoxifen injection, for long‐term adenoma studies, mice were sacrificed when they started to lose weight (more than 20% weight loss compared with the highest measured weight), or when mice appeared moribund due to other characteristics associated with adenoma development.

### Tissue processing and clone size quantifications

After the termination of all *in vivo* experiments, mice were dissected, and intestines were isolated and processed for further analysis. In short, intestines were cut open longitudinally and fixed in 4% paraformaldehyde (PFA) overnight at 4°C and kept in the dark. The next day, tissues were placed in 30% sucrose solution for 24 h at 4°C in order to preserve tissue integrity, after which tissues were frozen and stored at −80°C. Tissues were sliced using a Cryostar NX70 cryostat and carefully placed on glass slides. To determine the clone sizes of *Apc*‐mutant cells, RNA *in situ* hybridization (RNA‐ISH) was used to detect the presence of *Notum* mRNA according to a previously optimized protocol (van Neerven *et al*, 2021), slides were counterstained with hematoxylin, and mutant (*Notum* ^*+*^) clone sizes were quantified as proportions of the crypt circumference (1:8–8:8), and completely full (8:8) *Notum* ^*+*^ crypts are considered to be fixed by *Apc*‐mutant cells. Both adenomas and clone sizes were scored blindly.

### Human tissue collection

We collected fresh paired biopsies from adenomas and the surrounding macroscopically normal‐appearing intestinal epithelium from 61 patients undergoing routine colectomy. The collection of material was approved by the medical ethical committee of the Amsterdam UMC under approval number (2015–206), and all patients provided written informed consent. Biopsies were dissociated using the FastPrep‐24‐5G (MP Biomedicals) in combination with Lysis Tubes S (Qiagen) for two times 1 min at 6.5/s with 1 min on ice in between, material was collected using the Allprep DNA/RNA Universal kit (Qiagen). The isolation and maintenance of human colon organoids were described before (van Neerven *et al*, 2021). Collection of material was approved by the Medical Ethical Committee of the Amsterdam AMC under approval numbers 2014–178 and 09–146 with written informed consent of the patients, and the experiments conformed to the principles set out in the WMA Declaration of Helsinki and the Department of Health and Human Services Belmont Report.

### Organoid cultures

Mouse proximal small intestinal crypts were isolated from *Villin‐Cre* ^*ERT2*^;*Apc* ^*fl*/*fl*^ mice as previously described (Sato *et al*, 2009), grown in Matrigel (Corning), and cultured in DMEM/F12 medium supplemented with N2, B27, Glutamax, Hepes (5 mM), Antibiotic/Antimycotic (all Gibco), and N‐acetyl‐L‐cysteine (1 mM, Sigma). The culture medium was freshly supplemented with mouse EGF (50 ng/ml, TEBU‐BIO), R‐spondin1, and Noggin (both conditioned media), hereafter referred to as “ENR medium.” *Apc*‐mutant (*Apc* ^−/−^) organoids were cultured in the same medium but in the absence of R‐spondin1, or “EN medium.” To recombine the LoxP flanked *Apc* alleles, organoids were seeded and incubated with 1 μM of 4OH‐tamoxifen (Sigma) overnight, the next day medium was replaced for fresh ENR medium. Quantification of organoid growth was performed by bright‐field microscopic assessment of individual organoids that were cultured in the presence or absence of BVDU. At each timepoint, from day 1 to day 4 after seeding, images were taken and the mean organoid area of a minimum of 150 organoids per timepoint was quantified using ImageJ software. To generate *Hspb1* CRISPR knockout (KO) lines, unrecombined *Villin‐Cre* ^*ERT2*^;*Apc* ^*fl*/*fl*^ organoids were transduced with lentiviral particles generated from LentiCRISPR‐V2 plasmid (#52961, Addgene), by spinfection in the presence of 5 μM CHIR99021 (Axon Medchem), 10 μM ROCK inhibitor (Sigma), and 8 μg/ml Polybrene (Sigma) as previously described (van Neerven *et al*, 2022). To select for integration of the plasmid, organoids were incubated for 3 days in the presence of 1 μg/ml puromycin (InvivoGen), expanded, and sorted into 96‐well plates to generate single‐cell clones. Editing of the *Hspb1* gene was confirmed by Sanger sequencing. For *in vitro* inhibition of HSP25, BVDU (60 μM) was added to the medium immediately after plating the organoids. For heat shock experiments, equal numbers of (control or treated) organoids were harvested, incubated at 37°C (control) or 42.5°C (heat shock) for 60‐min in 15 ml tubes containing ENR medium before replated in fresh matrigel covered with fresh ENR medium, and clonogenic outgrowth was determined 4 days after replating. For all organoid experiments, medium was refreshed every other day. All organoids were routinely screened for mycoplasma.

### TCF4 assay

The maintenance and applications of the doxycycline‐inducible dominant negative TCF4 (dnTCF4) reporter cell lines have been described in detail elsewhere (Van de Wetering *et al*, 2002). For this study, we used the Ls174T colorectal cancer cell line (RRID:CVCL 1384), which is routinely cultured in DMEM/F12 (Gibco) supplemented with 10% FCS (Serana), 1% penicillin/streptomycin (Gibco), and 100× L‐glutamine (Gibco). In order to inactivate endogenous TCF4 and inhibit TCF4‐related transcription, cells were cultured in the presence of 1 μg/ml doxycycline (Sigma) for 48 h before RNA or protein was extracted. Ls174T cells were authenticated using STR profiling and routinely screened for mycoplasma.

### Generation of CRISPR constructs

To generate CRISPR KO organoids, three distinct sgRNA's were designed using Benchling software, aimed at targeting either exon1 or exon2 of the *Hspb1* gene. Sequences are sgRNA1: 5′ CGGTTGCCCGATGAGTGGTC 3′, sgRNA2: 5′ CTTCGCTCCGGAGGAGCTCA 3′, and sgRNA3: 5′ GAAGAAAGGCAGGACGAACA 3′. sgRNAs are cloned into the LentiCRISPR‐V2 plasmid (#52961, Addgene), and transformed into *Stabl3* competent bacteria (Invitrogen). Sanger sequencing was performed to check for correct insertion of sgRNAs, and lentiviral particles were subsequently generated as previously described (van Neerven *et al*, 2022).

### RNA extraction and RT‐qPCR analysis

For analysis of gene expression by qPCR, RNA was isolated using the Nucleospin RNA isolation kit (#740955, Bioke). cDNA was synthesized using SuperScript III RT (Sigma), and RT‐qPCRs were performed on the LightCycler 480 system using SYBR Green (both Roche). To analyze gene expression levels, the ΔΔCt method was applied, and all values were normalized to expression of housekeeping genes *Hprt* and *Rpl37* (mouse) or *GAPDH* and *GUSB* (human). The following primers were used for RT‐qPCR:

**Table:** 

Gene	Species	Sequence (5′–3′)
*Hspb1*	Mouse	FW: TCACCCGGAAATACACGCTC
		RV: GGCCTCGAAAGTAACCGGAA
*Axin2*	Mouse	FW: CCATGACGGACAGTAGCGTA
		RV: CTGCGATGCATCTCTCTCTG
*Lgr5*	Mouse	FW: TTCGTAGGCAACCCTTCTCT
		RV: TCCTGTCAAGTGAGGAAATTCA
*Notum*	Mouse	FW: CTGCGTGGTACACTCAAGGA
		RV: CCGTCCAATAGCTCCGTATG
*Hprt*	Mouse	FW: TGTAATGATCAGTCAACGGGGG
		RV: AGAGGTCCTTTTCACCAGCAA
*Rpl37*	Mouse	FW: CCAAGGCCTACCACCTTCAG
		RV: CAGTCCCGGTAGTGTTTCGT
*AXIN2*	Human	FW: CTCCTTATCGTGTGGGCAGT
		RV: CTTCATCCTCTCGGATCTGC
*P21*	Human	FW: AGTCAGTTCCTTGTGGAGCC
		RV: CATGGGTTCTGACGGACAT
*HSPB1*	Human	FW: GCGGAAATACACGCTGCCC
		RV: GACTCGAAGGTGACTGGGATG
*GAPDH*	Human	FW: AATCCCATCACCATCTTCCA
		RV: TGGACTCCACGACGTACTCA
*GUSB*	Human	FW: TGGTTGGAGAGCTCATTTGGA
		RV: GCACTCTCGTCGGTGACTGTT

### Microarray experiment and analysis

Microarray data used in this study were generated and published before (Smit *et al*, 2020a) and are publicly available through the National Center for Biotechnology Information (NCBI) Gene Expression Omnibus (GEO) under accession number GSE143509. In short, to capture the transcriptional profiles of *Apc*‐mutant organoids on the verge of transformation, *Villin‐Cre* ^*ERT2*^ *(wild type*, *WT)* and *Villin‐Cre* ^*ERT2*^;*Apc* ^*fl*/*fl*^ organoids were plated, and recombination was activated using 1 μM of 4OH‐tamoxifen. After 4 days, organoids were passaged to ensure full recombination, and RNA was isolated 3 days later using the ISOLATE II RNA Mini Kit (BIO‐52073, Bioline). A total of 400 ng of purified RNA was amplified and labeled using the 3′ IVT Pico Kit (Affymetrix) and RNA Amplification Kit (Nugene) according to manufacturer's protocols. Microarray analysis of mouse organoids was performed using Affymetrix Clariom S mouse array by the Dutch Genomics Service and Support Provider (MAD, Science Park, University of Amsterdam, The Netherlands). Washing and staining were performed by the GeneChip Fluidics Station 450, and the scanning was performed using the GeneChip Scanner 3000 7G (both Thermo Fisher Scientific). Data normalization, statistical testing, and extraction of differentially expressed genes were performed using the R2 platform (R2 R2 database, 2019).

### RNA‐ISH

RNA‐ISH was performed on both fixed‐frozen and paraffin embedded mouse organoids and tissues using the RNAscope 2.5 HD‐Brown kit (ACD Bio) according to manufacturer's protocols. RNAscope was used for the detection of *Notum* (probe #428981), *Lgr5* (probe #312171), or *Hspb1* (probe #488361) mRNA, or for the positive control *Ppib* (probe #313911), and slides were counterstained with hematoxylin.

### Digital droplet PCR

To quantitatively assess the amount of unrecombined (*Apc* ^fl/fl^) and recombined (*Apc* ^−/−^) *Apc* alleles in intestinal crypt fractions, we applied digital droplet PCR using the QX200 Droplet Digital PCR System (Bio‐Rad) in combination with EvaGreen supermix (#1864034, Bio‐Rad). Primers used to detect these *Apc* alleles are “*Apc* common” FW 5′ GTTCTGTATCATGGAAAGATAGGTGGTC 3′, “*Apc* ^fl/fl^” RV 5′ CACTCAAAACGCTTTTGAGGGTTG 3′, and “*Apc* ^−/−^” RV 5′ GAGTACGGGGTCTCTGTCTCAGTGAAG 3′.

### EdU assays

EdU incorporation assays were performed to assess the hyperproliferative phenotype of *Apc*‐mutant cells *in vivo*. EdU was administered 2 h before mice were sacrificed and tissues were immediately fixed in 4% PFA. For the visualization of proliferating cells, 10 μM thick cryosections were cut and processed for analysis using the EdU Click‐IT imaging kit (Thermo Fisher, C10234) according to the manufacturer's protocol. Cells were counterstained using Hoechst‐33342 and visualized using an SP8 confocal microscope (Leica).

### Immunofluorescence

Stainings were performed on intact, fixed mouse organoids. Organoids were harvested at designated timepoints together with surrounding Matrigel, carefully resuspended in Cell Recovery Solution (Corning), and incubated for 30 min on ice. After centrifugation, supernatant was removed and pellets were washed once in 1%FCS/PBS, before getting fixed in 4% PFA for 10 min at room temperature, in the dark. Organoids were permeabilized using 0.1% Triton X‐100 in PBS, for 15 min at room temperature, whereafter they were incubated overnight with anti‐HSP25 protein (Enzo Life Sciences, ADI‐SPA‐801‐D, RRID:AB_1193481, 1:100) dissolved in antibody diluent (ScyTek) at 4°C. The next day, samples were washed twice and incubated with anti‐Rabbit‐Alexa647 (Thermo, A‐21244, RRID:AB 2535812, 1:500) for 1 h at room temperature, protected from light. Samples were washed twice, and the actin cytoskeleton was stained using ActinGreen (Thermo Fisher, R37110), whereafter samples were washed again and counterstained with Hoechst‐33342 for 5 min. Next, samples were mixed with prolong, placed on microscopic slides, and covered with glass coverslips.

### Western blot

Protein lysates were prepared using RIPA Buffer (Thermo Fisher) and Halt protease inhibitor (Thermo Fisher) and incubated on ice for 10 min. Next, samples were centrifuged at 6,000 *g* for 10 min at 4°C, and the lysate (supernatant) was collected. Protein quantification was performed using the Pierce BCA protein assay kit (Thermo). After protein quantification, 30 μg lysates were prepared in 4× leammli sample buffer (Bio‐Rad), incubated for 5 min at 95°C, and centrifuged at 6,000 *g* for 10 min at 4°C. Protein samples were loaded into 4–15% pre‐cast gels (Bio‐Rad) and run on Mini‐PROTEAN Tetra system (Bio‐Rad). Gels were blotted on PVDF membranes (Bio‐Rad) using the TransBlot Turbo system (Bio‐Rad), blocked in 5% skim milk powder (Sigma) for 1 h, and washed in 1× TBST. Blots were incubated with primary antibodies in 5% BSA (Fitzgerald Industries) overnight at 4°C. The next day, blots were washed, incubated with secondary antibody for 1 h at room temperature. After incubation, blots were thoroughly washed, and proteins were visualized using Pierce ECL Western Blotting substrate (Thermo) according to manufacturer's protocol.

Primary antibodies are anti‐HSP25 (Enzo Life Sciences, ADI‐SPA‐801‐D, RRID:AB_1193481, 1:1,000), anti‐HSP27 (Santa Cruz, sc‐13132, RRID:AB 627755 1:1,000), anti‐HSPB8 (CST, 3059 S, RRID:AB 2248643, 1:1,000), anti‐DNAJB6 (Abcam, ab198995, 1:1,000), anti‐GAPDH (Millipore, MAB374, RRID:AB 2107445 1:1,000). Secondary antibodies are anti‐mouse‐HRP (Southern Biotech, 1070‐05, RRID:AB 2650509 1:10,000), anti‐rabbit‐HRP (Cell Signaling, 7074, RRID:AB 2099233, 1:5,000).

### Statistical analysis

Visualization of the data and statistical analyses were performed using GraphPad Prism. For every experiment, the statistical test used is noted in the figure legends, significant results are represented with asterisks, *P ≤ 0.05, **P ≤ 0.01, ***P ≤ 0.001, ****P ≤ 0.0001, ns, not significant. Exact *P*‐values are displayed in the figure legends. For all, sample sizes were determined based on previous studies with a comparable study design (Vermeulen *et al*, 2013; Van Der Heijden *et al*, 2016; van Neerven *et al*, 2021).

## Author contributions

**Sanne M van Neerven:** Conceptualization; data curation; formal analysis; supervision; validation; investigation; visualization; methodology; writing – original draft; writing – review and editing. **Wouter L Smit:** Conceptualization; data curation; formal analysis; investigation; methodology; writing – review and editing. **Milou S van Driel:** Investigation. **Vaishali Kakkar:** Conceptualization; investigation. **Nina E de Groot:** Investigation. **Lisanne Nijman:** Investigation. **Clara C Elbers:** Funding acquisition. **Nicolas Léveillé:** Supervision. **Jarom Heijmans:** Conceptualization; supervision. **Louis Vermeulen:** Conceptualization; supervision; funding acquisition; validation; writing – original draft; writing – review and editing.

## Disclosure and competing interests statement

LV received consultancy fees from Bayer, MSD, Genentech, Servier, and Pierre Fabre, but these had no relation to the content of this publication.

**The paper explained**

**Problem**

Chemoprevention strategies for high‐risk CRC patients such as *familial adenomatous polyposis* (FAP) patients, who carry a heterozygous germline mutation in tumor suppressor gene *APC*, are currently lacking. Upon a second genetic hit in *APC*, the master tumor suppressor gene in most colorectal cancers (CRCs), intestinal epithelial cells rapidly undergo oncogenic transformation. These transformed cells give rise to excessive polyp formation, leading to a 100% risk of CRC development in these patients. Therefore, mechanistic insight and means to prevent oncogenic transformation are needed.

**Results**

We set out to capture the transcriptomic profiles of *Apc*‐mutant intestinal epithelial organoids during transformation and observed marked upregulation of a set of genes involved in protein folding and buffering of proteotoxic stress, in particular the small heat shock protein and chaperone HSP25, a mouse ortholog of human HSP27. Both genetic deletion and chemical inhibition *in vitro* studies revealed that clonal expansion of *Apc*‐mutant organoids was strongly dependent on HSP25, without affecting wild‐type organoids. To translate these findings to an *in vivo* setting, we conditionally deleted *Apc* in two distinct mouse models and demonstrated that oral administration of HSP25 inhibitor brivudine, a commonly used antiviral drug, significantly impaired adenoma formation. Critically, we also observed elevated HSP27 expression in human adenomas and in human FAP organoids and demonstrated that these FAP organoids were also sensitive to HSP27 inhibition.

**Impact**

This study reveals HSP25/27 as a specific vulnerability to transforming *APC*‐mutant cells, due to its role in channeling proteotoxic stress associated with oncogenic transformation. We propose HSP27 as a novel therapeutic target for chemoprevention strategies in patients at a high risk of developing *APC‐*driven CRCs such as FAP patients.

## Supporting information



Expanded View Figures PDF

Source Data for Expanded View

Source Data for Figure 1

Source Data for Figure 2

Source Data for Figure 3

PDF+

## Data Availability

This study includes no data deposited in external repositories.
