# Supplementary material for: Intestinal Apc‐inactivation induces HSP25 dependency
Source: EMBO Mol Med. 2022 Nov 2;14(12):e16194. doi: 10.15252/emmm.202216194 (PMC9727927; doi:10.15252/emmm.202216194)
Supplement: Supplementary file 1 — Expanded View Figures PDF [file EMMM-14-e16194-s005.pdf]

Expanded View Figures

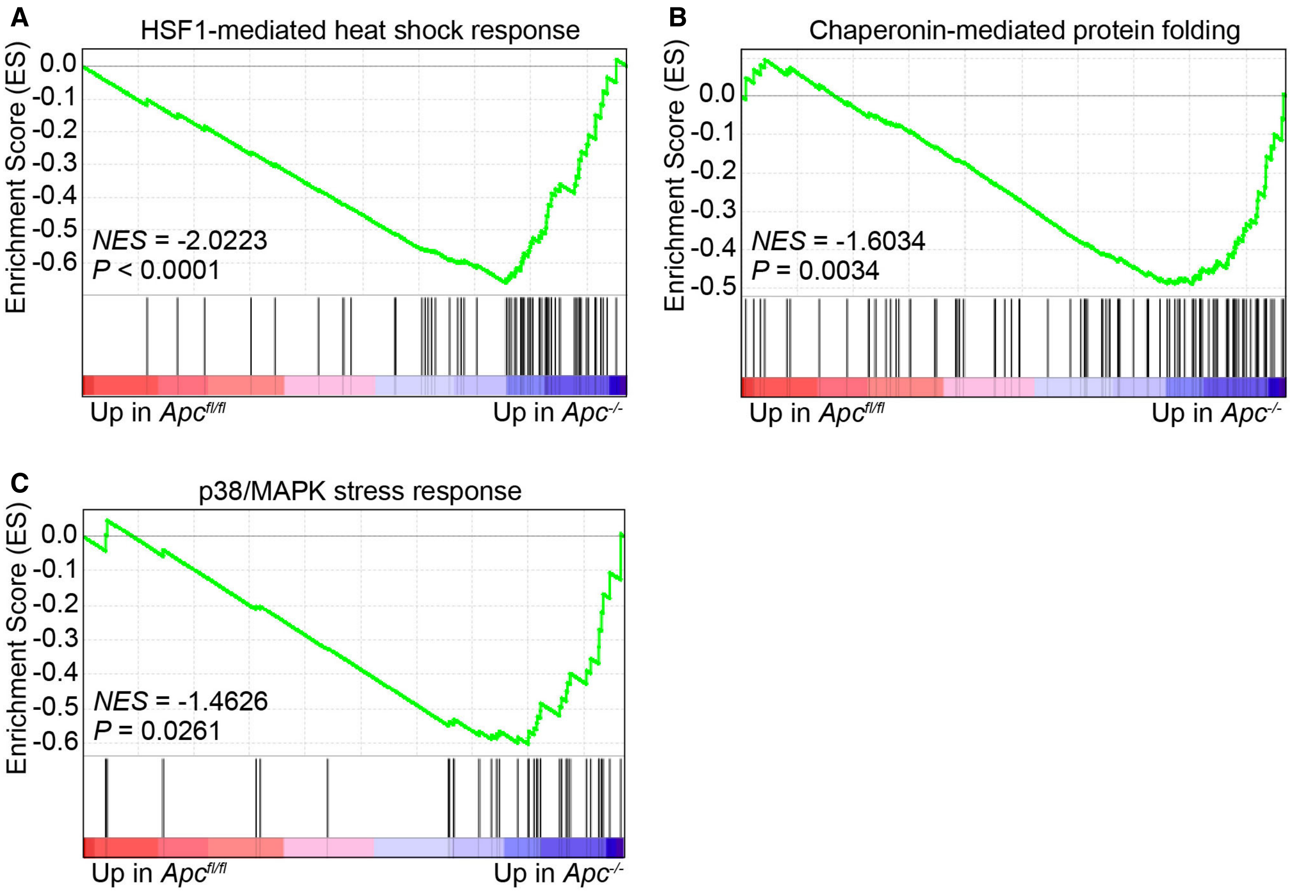

Figure EV1. Loss of Apc induces cellular stress.

A–C Gene Set Enrichment Analysis for pathways involved in HSF1-mediated heat shock response (A), chaperonin-mediated protein folding (B), and p38/MAPK stress response (C). NES, Normalized Enrichment Score; *P*, nominal *P*-value of the enrichment score, which is based on a phenotype-based permutation test procedure as described in more detail in (Subramanian *et al*, 2005).

Source data are available online for this figure.

 © 2022 The Authors

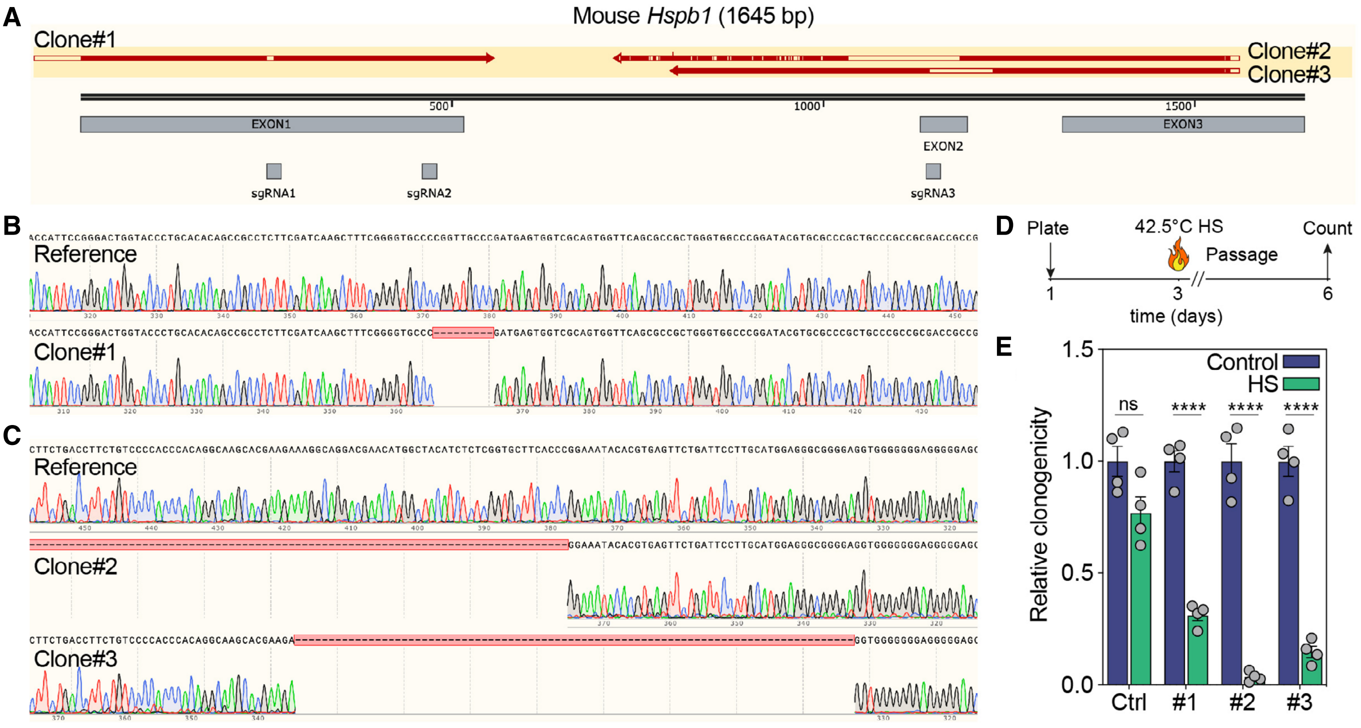

Figure EV2. Validation of Hspb1 KO clones.

A Overview of Hspb1 gene, positions of the sgRNA's and edited sites within the KO clones.

B, C Sanger sequencing results of the edited regions in exon 1 (B) or exon 2 (C).

D, E Illustration of the heat shock (HS) clonogenicity experiment (D), and quantification of clonogenicity in KO clones in untreated or HS-treated conditions (****$P < 0.0001$ for KO#1, 2, and 3, $n = 4$ experiments, unpaired two-sided t-test).

Source data are available online for this figure.

Figure EV3. Effect of *in vitro* HSP25 inhibition using BVDU.

A Growth curves of Apc-mutant organoids.

B–D Growth curves of WT organoids (B), representative images of WT organoids cultured in the absence or presence of 60 μM BVDU (C), and quantification of their clonogenic potential (D). Scale bar, 500 μm.

E RNA-ISH for Lgr5 in control or BVDU-treated WT organoids. Scale bar, 50 μm, zoom panel 20 μm.

F, G Representative images (F) and clonogenicity (G) of WT organoids treated with CHIR99021 in the absence or presence of BVDU. (****$P < 0.0001$, $n = 4$). Scale bar, 500 μm.

H, I Relative Hspb1 (H) and HSP25 (I) expression in wild-type organoids after heat shock (HS) treatment (**$P = 0.0016$).

J–L Illustration of the heat shock (HS) clonogenicity experiment (J), representative images of control or BVDU-treated WT organoids after HS treatment (K), and quantification of clonogenicity (*$P = 0.0128$) (L).

Data information: All data are mean ± s.e.m., $n = 3$ biological replicates, analyzed using unpaired two-sided t-test.
Source data are available online for this figure.

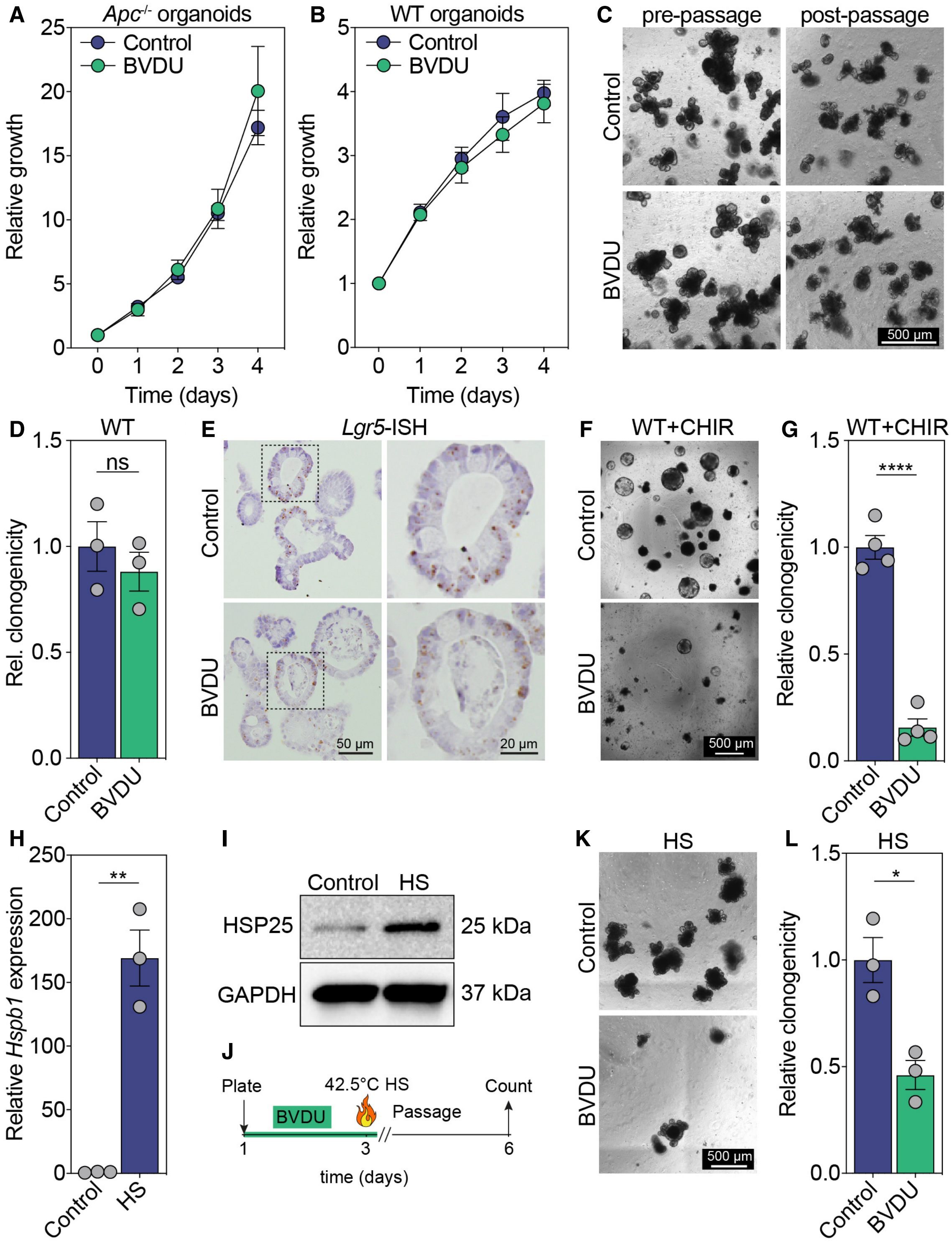

Figure EV3.

© 2022 The Authors

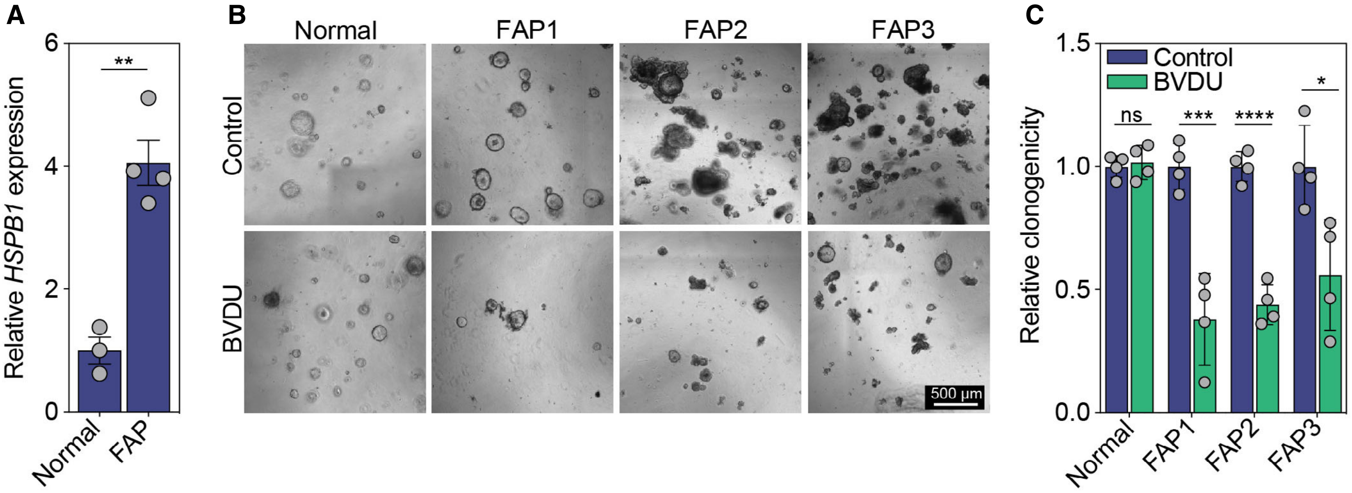

Figure EV4. Effect of BVDU on human FAP organoids.

A Relative HSPB1 expression in normal colon organoids (n = 3) and organoids derived from patients with familial adenomatous polyposis (**P = 0.0013, n = 4, mean ± s.e.m.).

B, C representative images of control or BVDU-treated human organoids (B) and quantification of clonogenicity (C) (***P = 0.0009 (FAP1), ****P < 0.0001 (FAP2), *P = 0.0197 (FAP3), n = 4 wells per line, data are mean ± s.d.) scale bar, 500 μm.

Data information: All data are analyzed using unpaired two-sided t-test.
Source data are available online for this figure.

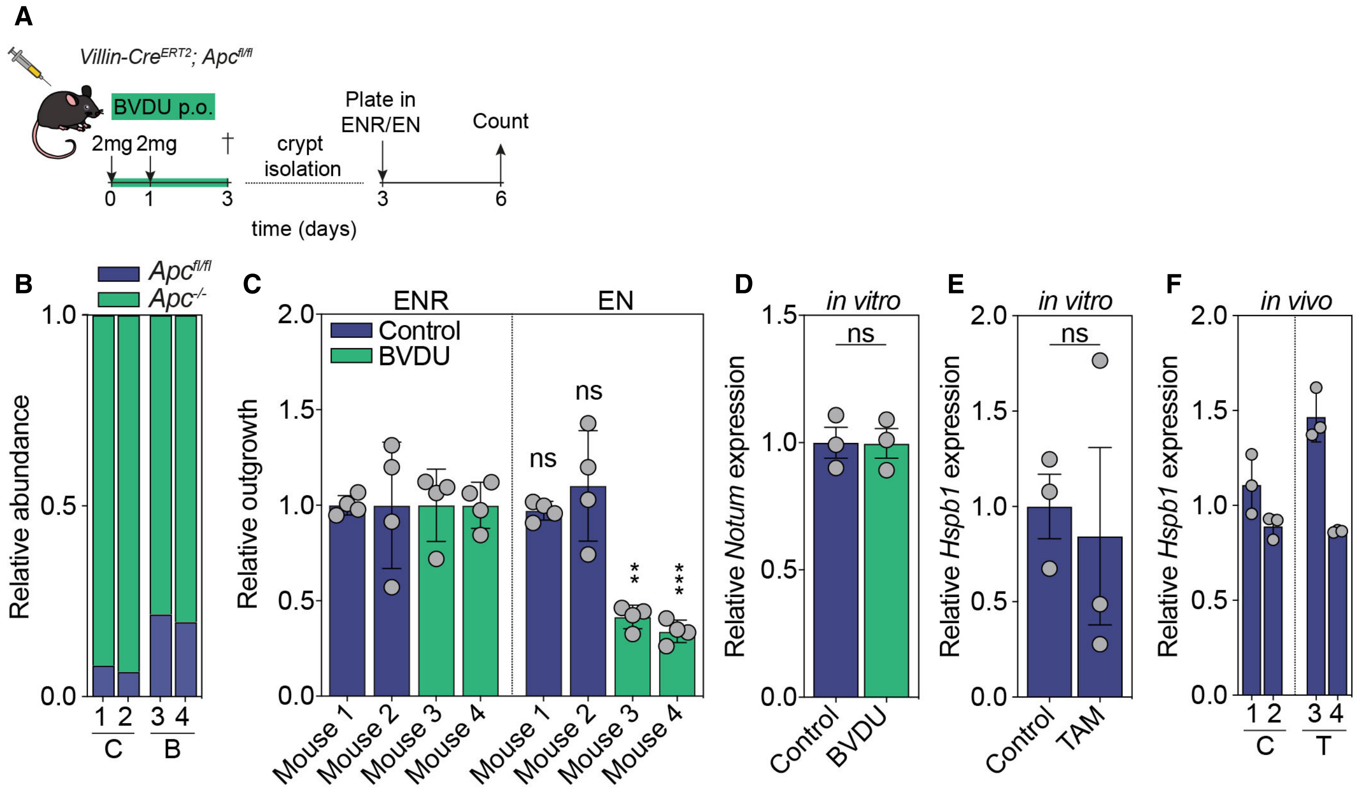

Figure EV5. Validation of *in vivo* BVDU treatment.

A Illustration of experimental setup for crypt isolation.

B Ratio of unrecombined versus recombined Apc alleles in control and BVDU treated crypts (n = 2 mice per group, C, control, B, BVDU treated).

C Outgrowth of crypts isolated from control and BVDU-treated mice in ENR (mEGF, Noggin, and Rspondin1) or EN medium (mEGF, Noggin) (**P = 0.0010 (mouse 3), ***P < 0.0001 (mouse 4), n = 4 wells per condition, data are mean ± s.d.).

D Relative Notum expression in control or BVDU-treated Apc organoids (n = 3 experiments).

E, F Relative Hspb1 expression in control or tamoxifen-treated organoids (n = 3 experiments) (E) or intestinal tissues (F) (n = 2 mice per condition, n = 3 technical replicates, C, control, T, Tamoxifen).

Data information: All data are mean ± s.e.m., unless otherwise specified, analyzed using unpaired two-sided t-test.
Source data are available online for this figure.

© 2022 The Authors
