# Supplementary material for: Intestinal Apc‐inactivation induces HSP25 dependency
Source: EMBO Mol Med. 2022 Nov 2;14(12):e16194. doi: 10.15252/emmm.202216194 (PMC9727927; doi:10.15252/emmm.202216194)
Supplement: Supplementary file 2 — Source Data for Expanded View [file EMMM-14-e16194-s003.zip › Figure EV3/Western blots figure EV3.pptx]

## Slide 1
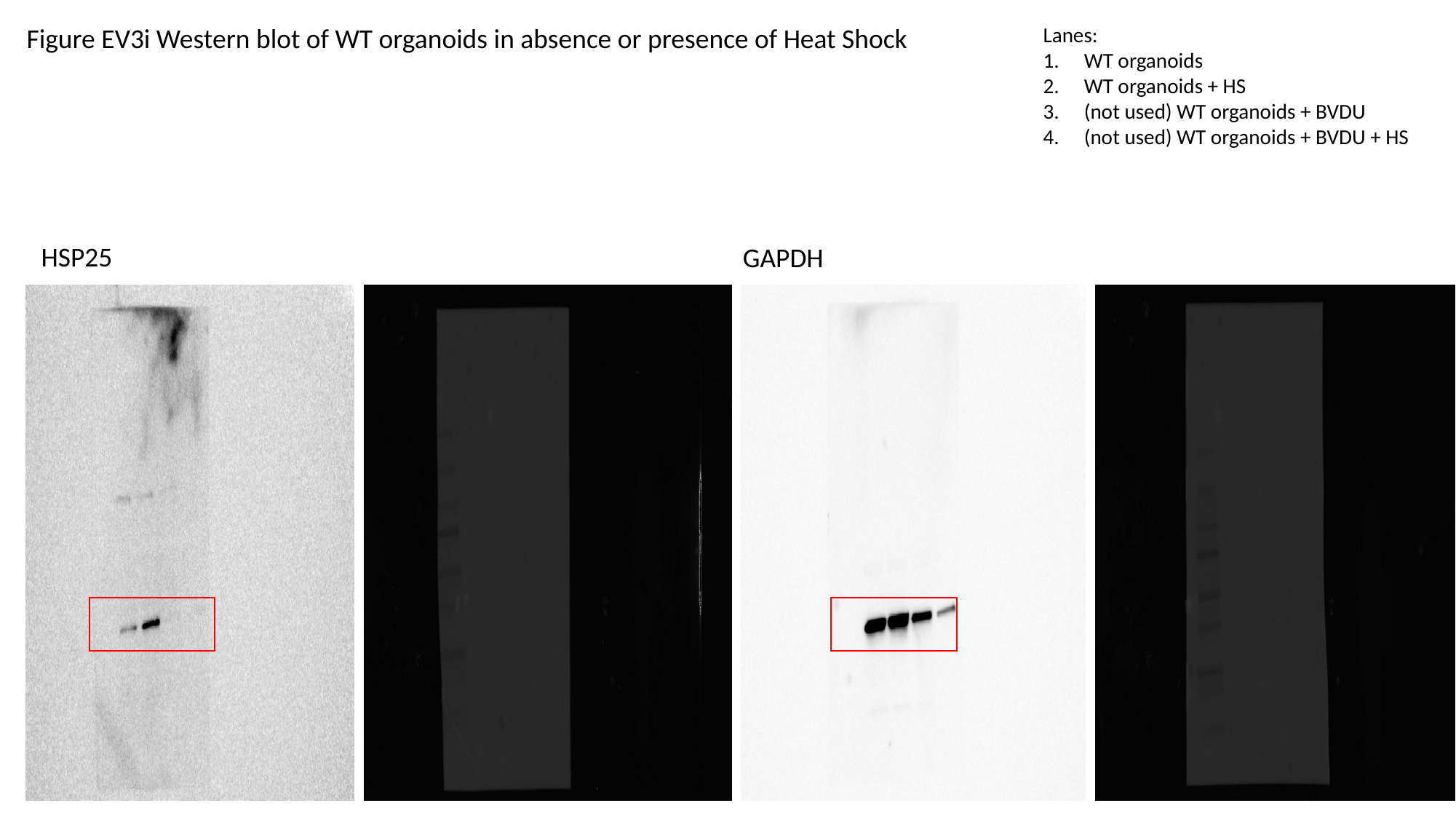

Figure EV3i Western blot of WT organoids in absence or presence of Heat Shock
Lanes:
WT organoids
WT organoids + HS
(not used) WT organoids + BVDU
(not used) WT organoids + BVDU + HS
HSP25
GAPDH
