# Supplementary material for: Intestinal Apc‐inactivation induces HSP25 dependency
Source: EMBO Mol Med. 2022 Nov 2;14(12):e16194. doi: 10.15252/emmm.202216194 (PMC9727927; doi:10.15252/emmm.202216194)
Supplement: Supplementary file 4 — Source Data for Figure 2 [file EMMM-14-e16194-s007.zip › Figure 2/Western blots figure 2.pptx]

## Slide 1
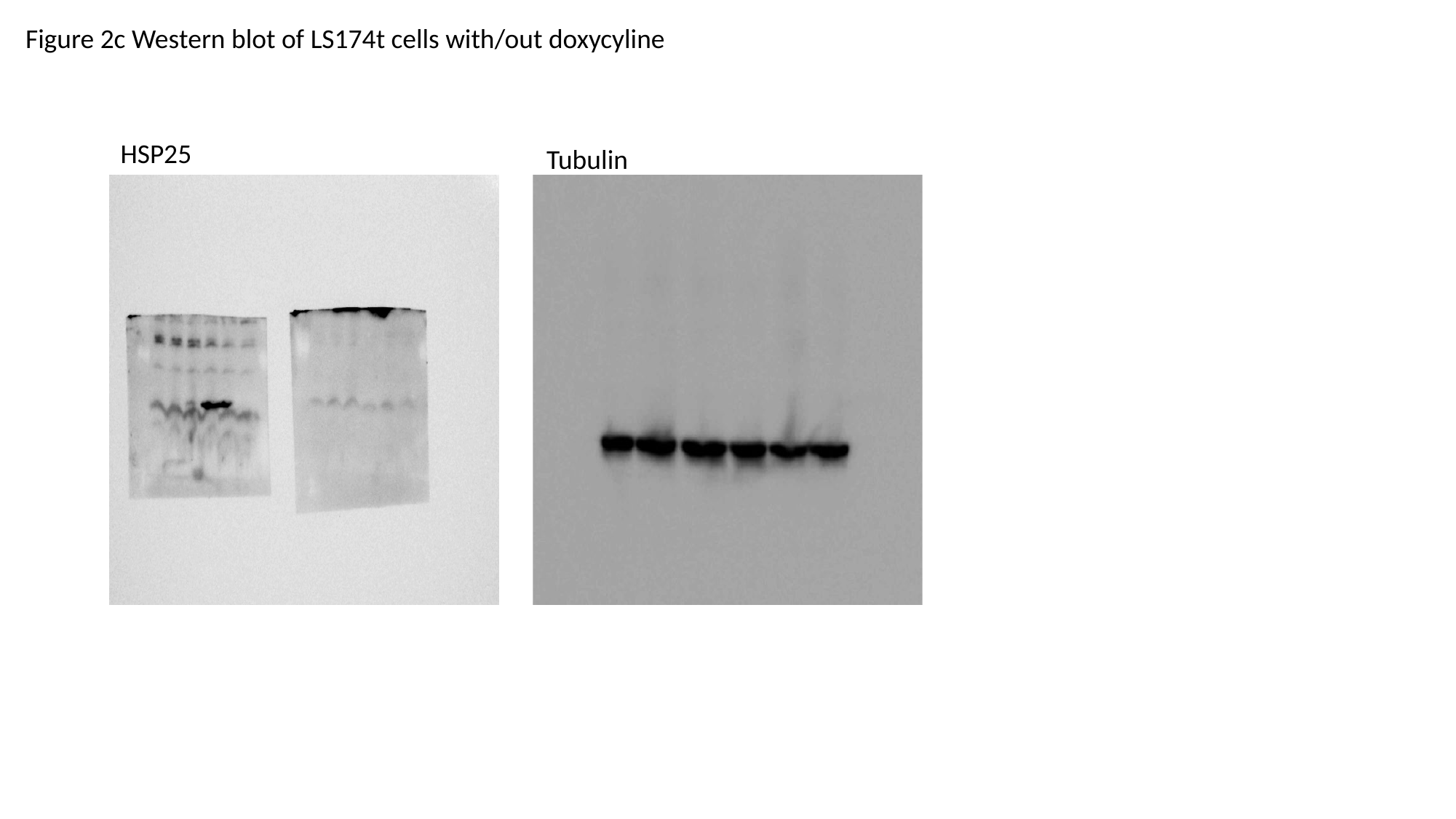

Figure 2c Western blot of LS174t cells with/out doxycyline
HSP25
Tubulin

## Slide 2
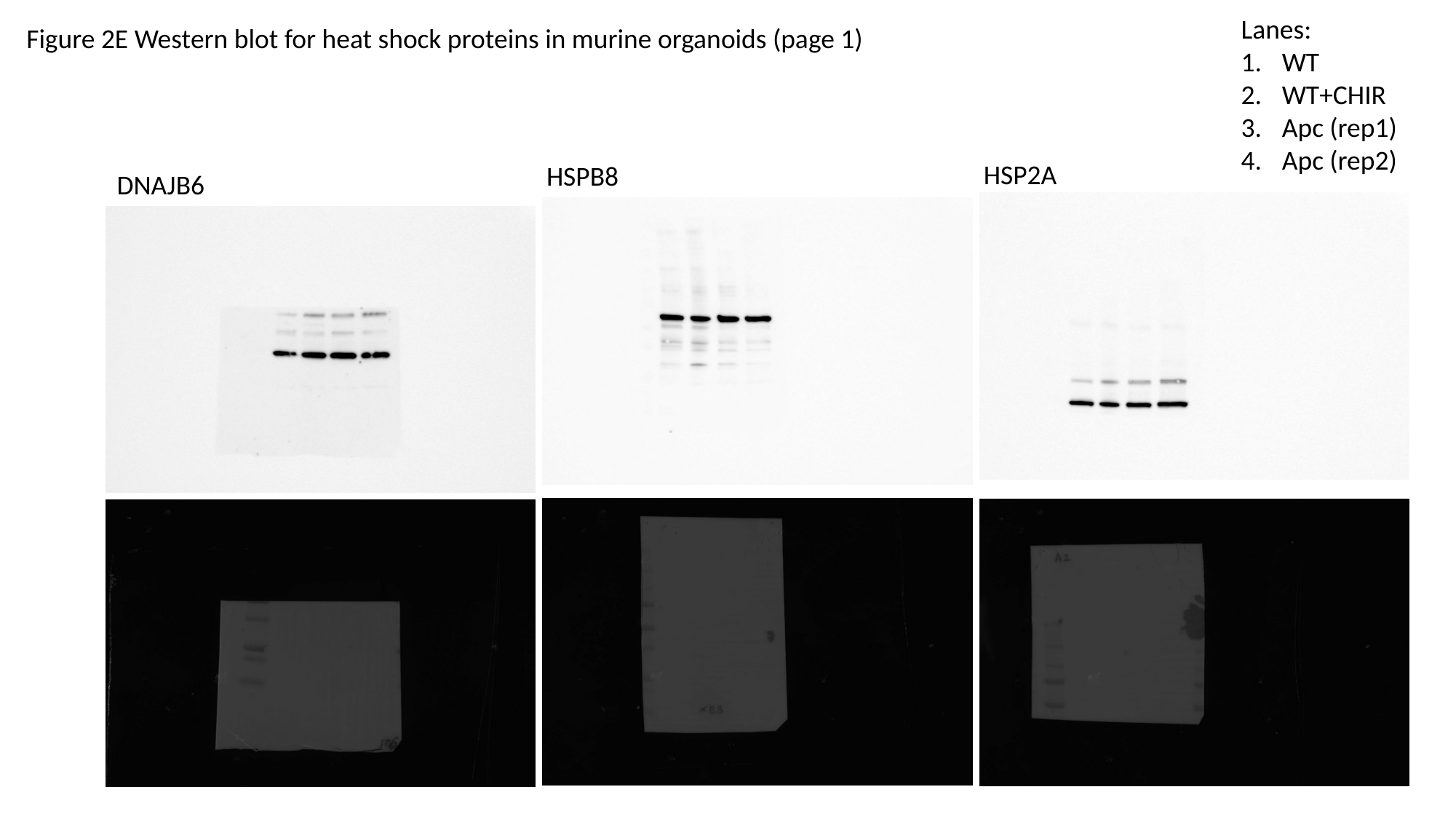

Lanes:
WT
WT+CHIR
Apc (rep1)
Apc (rep2)
Figure 2E Western blot for heat shock proteins in murine organoids (page 1)
HSP2A
HSPB8
DNAJB6

## Slide 3
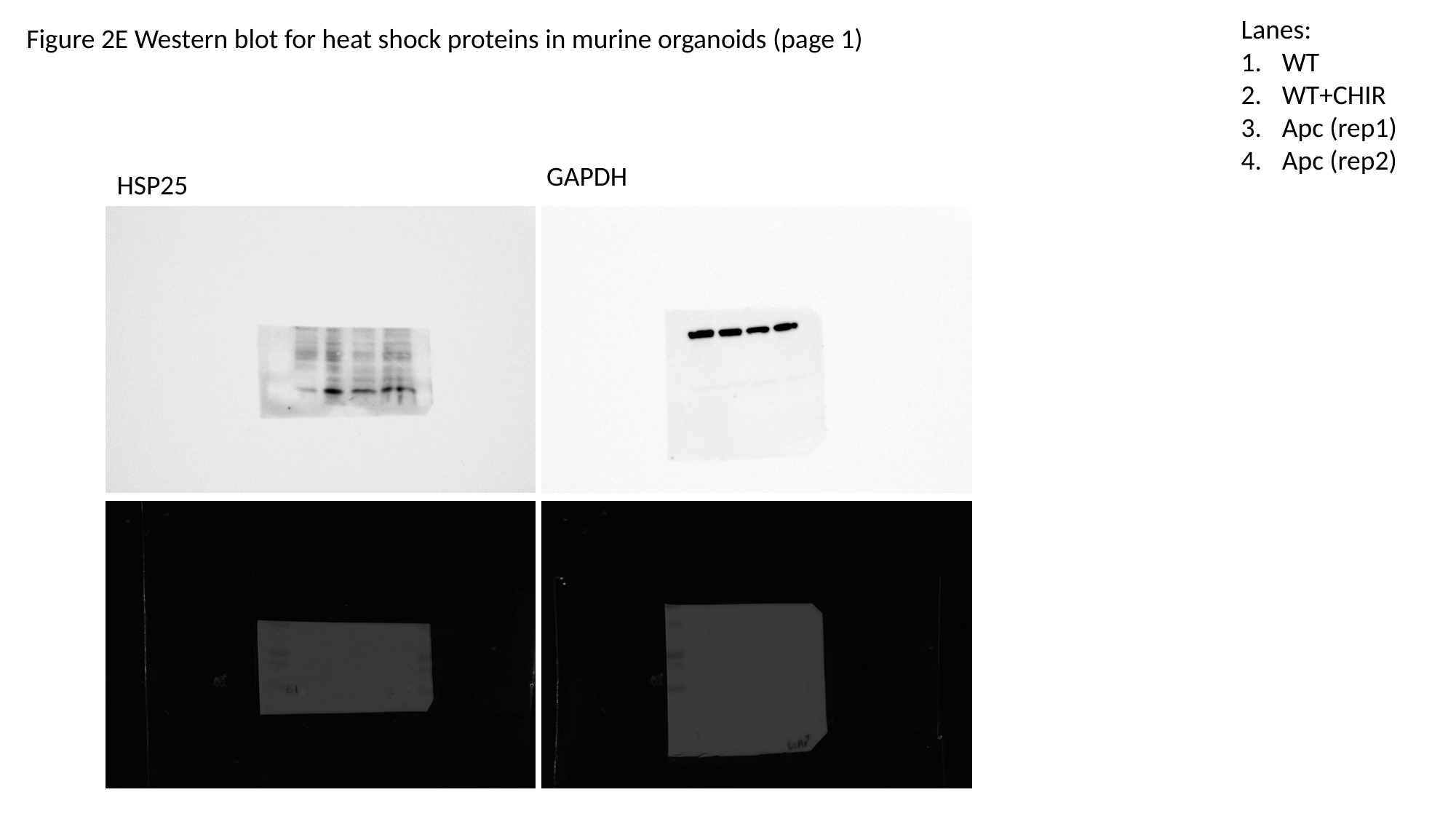

Lanes:
WT
WT+CHIR
Apc (rep1)
Apc (rep2)
Figure 2E Western blot for heat shock proteins in murine organoids (page 1)
GAPDH
HSP25
