# Supplementary material for: Intestinal Apc‐inactivation induces HSP25 dependency
Source: EMBO Mol Med. 2022 Nov 2;14(12):e16194. doi: 10.15252/emmm.202216194 (PMC9727927; doi:10.15252/emmm.202216194)
Supplement: Supplementary file 7 [file EMMM-14-e16194-s001.pdf]

EMBO Molecular Medicine

Intestinal Apc-inactivation induces HSP25 dependency

Sanne van Neerven, Wouter Smit, Milou van Driel, Vaishali Kakkar, Nina de Groot, Lisanne Nijman, Clara Elbers, Nicolas Léveillé, Jarom Heijmans, and Louis Vermeulen

DOI: 10.15252/emmm.202216194

Corresponding authors: Louis Vermeulen (l.vermeulen@amsterdamumc.nl) , Sanne van Neerven (s.m.vanneerven@amsterdamumc.nl)

Review Timeline:

Submission Date:	19th Apr 22
Editorial Decision:	18th May 22
Revision Received:	23rd Aug 22
Editorial Decision:	7th Oct 22
Revision Received:	12th Oct 22
Accepted:	12th Oct 22

Editor: Lise Roth

Transaction Report:

(Note: With the exception of the correction of typographical or spelling errors that could be a source of ambiguity, letters and reports are not edited. Depending on transfer agreements, referee reports obtained elsewhere may or may not be included in this compilation. Referee reports are anonymous unless the Referee chooses to sign their reports.)

18th May 2022

Dear Prof. Vermeulen,

Thank you for the submission of your manuscript to EMBO Molecular Medicine. We have now received feedback from the three reviewers who agreed to evaluate your manuscript. As you will see from the reports below, the referees acknowledge the translational interest of the study, but also raise several points (mostly pertaining to the mechanistic insights) that should be addressed in a revised version of the current manuscript.

Addressing the reviewers' concerns in full will be necessary for further considering the manuscript in our journal, and acceptance of the manuscript will entail a second round of review. EMBO Molecular Medicine encourages a single round of revision only and therefore, acceptance or rejection of the manuscript will depend on the completeness of your responses included in the next, final version of the manuscript. For this reason, and to save you from any frustrations in the end, I would strongly advise against returning an incomplete revision.

Revised manuscripts should be submitted within three months of a request for revision; they will otherwise be treated as new submissions, except under exceptional circumstances in which a short extension is obtained from the editor.

When submitting your revised manuscript, please carefully review the instructions that follow below. We perform an initial quality control of all revised manuscripts before re-review; failure to include requested items will delay the evaluation of your revision.

We require:

1) A .docx formatted version of the manuscript text (including legends for main figures, EV figures and tables). Please make sure that the changes are highlighted to be clearly visible.

2) Individual production quality figure files as .eps, .tif, .jpg (one file per figure). For guidance, download the 'Figure Guide PDF' (https://www.embopress.org/page/journal/17574684/authorguide#figureformat).

3) A .docx formatted letter INCLUDING the reviewers' reports and your detailed point-by-point responses to their comments. As part of the EMBO Press transparent editorial process, the point-by-point response is part of the Review Process File (RPF), which will be published alongside your paper.

4) A complete author checklist, which you can download from our author guidelines (https://www.embopress.org/page/journal/17574684/authorguide#submissionofrevisions). Please insert information in the checklist that is also reflected in the manuscript. The completed author checklist will also be part of the RPF.

5) Please note that all corresponding authors are required to supply an ORCID ID for their name upon submission of a revised manuscript.

6) It is mandatory to include a 'Data Availability' section after the Materials and Methods. Before submitting your revision, primary datasets produced in this study need to be deposited in an appropriate public database, and the accession numbers and database listed under 'Data Availability'. Please remember to provide a reviewer password if the datasets are not yet public (see https://www.embopress.org/page/journal/17574684/authorguide#dataavailability).
In case you have no data that requires deposition in a public database, please state so in this section. Note that the Data Availability Section is restricted to new primary data that are part of this study.

7) For data quantification: please specify the name of the statistical test used to generate error bars and P values, the number (n) of independent experiments (specify technical or biological replicates) underlying each data point and the test used to calculate p-values in each figure legend. The figure legends should contain a basic description of n, P and the test applied. Graphs must include a description of the bars and the error bars (s.d., s.e.m.). Exact p values should be included.

8) We would also encourage you to include the source data for figure panels that show essential data. Numerical data should be provided as individual .xls or .csv files (including a tab describing the data). For blots or microscopy, uncropped images should be submitted (using a zip archive if multiple images need to be supplied for one panel). Additional information on source data and instruction on how to label the files are available at
.

9) Our journal encourages inclusion of *data citations in the reference list* to directly cite datasets that were re-used and obtained from public databases. Data citations in the article text are distinct from normal bibliographical citations and should directly link to the database records from which the data can be accessed. In the main text, data citations are formatted as

follows: "Data ref: Smith et al, 2001" or "Data ref: NCBI Sequence Read Archive PRJNA342805, 2017". In the Reference list, data citations must be labeled with "[DATASET]". A data reference must provide the database name, accession number/identifiers and a resolvable link to the landing page from which the data can be accessed at the end of the reference. Further instructions are available at .

10) We replaced Supplementary Information with Expanded View (EV) Figures and Tables that are collapsible/expandable online. A maximum of 5 EV Figures can be typeset. EV Figures should be cited as 'Figure EV1, Figure EV2" etc... in the text and their respective legends should be included in the main text after the legends of regular figures.
- For the figures that you do NOT wish to display as Expanded View figures, they should be bundled together with their legends in a single PDF file called *Appendix*, which should start with a short Table of Content. Appendix figures should be referred to in the main text as: "Appendix Figure S1, Appendix Figure S2" etc.
- Additional Tables/Datasets should be labeled and referred to as Table EV1, Dataset EV1, etc. Legends have to be provided in a separate tab in case of .xls files. Alternatively, the legend can be supplied as a separate text file (README) and zipped together with the Table/Dataset file.
See detailed instructions here:
.

11) The paper explained: EMBO Molecular Medicine articles are accompanied by a summary of the articles to emphasize the major findings in the paper and their medical implications for the non-specialist reader. Please provide a draft summary of your article highlighting
- the medical issue you are addressing,
- the results obtained and
- their clinical impact.

This may be edited to ensure that readers understand the significance and context of the research. Please refer to any of our published articles for an example.

12) For more information: There is space at the end of each article to list relevant web links for further consultation by our readers. Could you identify some relevant ones and provide such information as well? Some examples are patient associations, relevant databases, OMIM/proteins/genes links, author's websites, etc...

13) Conflict of interest: We updated our journal's competing interests policy in January 2022 and request authors to consider both actual and perceived competing interests. Please review the policy https://www.embopress.org/competing-interests and update your competing interests if necessary.

14) Every published paper now includes a 'Synopsis' to further enhance discoverability. Synopses are displayed on the journal webpage and are freely accessible to all readers. They include a short stand first (maximum of 300 characters, including space) as well as 2-5 one-sentences bullet points that summarizes the paper. Please write the bullet points to summarize the key NEW findings. They should be designed to be complementary to the abstract - i.e. not repeat the same text. We encourage inclusion of key acronyms and quantitative information (maximum of 30 words / bullet point). Please use the passive voice. Please attach these in a separate file or send them by email, we will incorporate them accordingly.

Please also suggest a striking image or visual abstract to illustrate your article as a PNG file 550 px wide x 300-600 px high.

15) As part of the EMBO Publications transparent editorial process initiative (see our Editorial at http://embomolmed.embopress.org/content/2/9/329), EMBO Molecular Medicine will publish online a Review Process File (RPF) to accompany accepted manuscripts.
In the event of acceptance, this file will be published in conjunction with your paper and will include the anonymous referee reports, your point-by-point response and all pertinent correspondence relating to the manuscript. Let us know whether you agree with the publication of the RPF and as here, if you want to remove or not any figures from it prior to publication.
Please note that the Authors checklist will be published at the end of the RPF.

EMBO Molecular Medicine has a "scooping protection" policy, whereby similar findings that are published by others during review or revision are not a criterion for rejection. Should you decide to submit a revised version, I do ask that you get in touch after three months if you have not completed it, to update us on the status.

I look forward to receiving your revised manuscript.

With kind regards,

Lise

Lise Roth, PhD
Senior Editor
EMBO Molecular Medicine

***** Reviewer's comments *****

Referee #1 (Remarks for Author):

The study by S.M. Van Neerven et al. aims to uncover the role of the protein chaperone HSP25 (HSP27 in humans) during Colorectal Cancer initiation. They used organoids and mouse genetics models of tumor initiation based on APC loss. The authors showed that HSP25 expression is induced in response to Wnt-β-catenin activation and facilitates adenoma transformation. This study describes a novel cancer vulnerability during initiation and therefore is of interest. However, there are important issues that must be addressed:

Major comments:

1- My main concern is the use of BVDU as a chemical inhibitor of HSP25:

- Authors must show functional inhibition of HSP25 by BVDU in their models.

- BVDU is a thymidine analog with viral specificity, but some of its metabolites inhibit endogenous cellular enzymes (and, for example, synergize with chemotherapy). As most experiments of in-vivo adenoma formation rely on chemical inhibition of HSP25 by BVDU, authors must provide evidence that observed therapeutic effects are due to inhibition of HSP25 activity.

2- A better characterization of Hspb1 KO clones is required. For example, the authors did not demonstrate a loss of HSP25 expression in the Hspb1 KO clones. It is also important to verify whether the effects of BVDU on oncogenic transformation are rescued in Hspb1 KO clones.

3- The possibility that Apc-mutant cells may depend on HSP25 to cope with proteotoxic stress is appealing. However, induction of proteotoxic stress should be addressed in the models used by the authors, as HSP25 also interacts with other clients involved in apoptosis or cytoskeleton remodeling.

4- Fig 3b: the authors must show HSP25 protein expression in the epithelium in the presence and absence of 4-OHT administration.

5- The authors observed a reduction of the Wnt-target genes LGR5 and Axin2 upon BVDU treatment. Do they recapitulate those findings in Hspb1 KO clones? Is it due to stem cell negative selection or to a role of HSP25 in regulating Wnt activity?

Minor Comments:

1- The authors state: "Together, these data indicate that loss of Apc and the subsequent activation of Wnt signaling directly drives the upregulation of Hspb1 in a TCF-dependent fashion". But, HSP25 is not necessarily a direct target of TCF/LEF, as MYC could also drive its expression. Please discuss this possibility.

2- Clone size quantification relies exclusively on Notum expression. It is advisable to confirm these results with another adenoma marker, as Notum might be a direct or indirect client of HSP25.

3- Can the authors explain how they measured "clone fixation"? It seems that these analyses were performed in a steady-state situation.

4- HSP27 has been associated with poor prognosis in various cancers. Can the authors discuss the potential role of HSP25 in established adenocarcinomas?

5- Please detail how organoid expansion was measured in Supplementary Figure 1 and provide data on BVDU-treated VillinCreERT2;Apc-/- organoids.

Referee #2 (Comments on Novelty/Model System for Author):

This is a highly relevant and novel study that uses state of the art in vitro (organdies) and in vivo (genetically engineered mice) models and techniques (genetic as well a pharmacological perturbation of intestinal adenomas/organoids). The data as presented are novel, sound and exciting, because they define a novel selective druggable target in APC mutant intestinal adenomas. This is of high clinical relevance for persons at risk for intestinal cancer formation, e.g. due to hereditary syndromes, such as APC mutant familial adenomatous polyposis (FAP).

Referee #2 (Remarks for Author):

In this study, van Neerven and colleagues investigated Wnt driven transcriptional programs during early intestinal cancer formation. During this process, they discovered strong induction of Hspb1, a heat shock protein, which is involved in protein refolding upon cellular stress, apoptosis inhibition, and cytoskeleton remodeling, processes known to be critically regulated by Wnt signalling. This suggests that Hspb1 is a critical executer of Wnt-driven cancer formation. In subsequent experiments, the authors validated this critical function in vitro in organoids and in vivo in mice genetically as well as via pharmacological inhibition. Finally, they show that blockade of Hspb1 specifically decreased the outgrowth Apc tumor cells, but not normal intestinal epithelial cells. This resulted in increased survival of Apc mutant mice.

This is an important study revealing a critical role of the chaperone Hsbp1 for intestinal cancer formation. The techniques used are state of the art and the manuscript is clearly written. The conclusions are supported by the data as presented. However, a few more data and experiments could significantly strengthen this manuscript.

Major concerns:
1) Mechanisms of Hsbp1 overexpression and its role in human colo-rectal cancer: The authors demonstrate convincingly that Hsbp1 is transcriptionally regulated by Wnt pathway activation. Is this the only route of Hsbp1 activation in intestinal cancer? This can be tested e.g. by mining public datasets of human adenomas/CRC for copy number variation/amplification of the Hsbp1 locus or hotspot mutations as well as the epigenetic regulation of Hsbp1.
2) Is Hsbp1 overexpression associated with a poor patient survival or other important clinical features, such as tumor differentiation and metastasis? Does BVDU treatment induces morphological changes or apoptosis of the adenomas?
3) What´s the mode of action of BVDU? More data on potential side effects (e.g. weight loss or other adverse effects of treated mice) and off target effects of BVDU would strengthen the translational impact of the manuscript.

Referee #3 (Comments on Novelty/Model System for Author):

The models used are appropriate and the technical quality is good. There is high potential for medical impact. The novelty is high for the model system being used but similar results have been reported in other systems. This doesn't detract from the overall potential impact of the work.

Referee #3 (Remarks for Author):

In this report Neerven and colleagues identify a dependency for Apc mutant cells on the heat shock protein HSP25. They outline a number of experiments detailing a transcriptional upregulation of Hspb1 (which encodes HSP25) following Apc loss and the requirement of Apc KO cells on its expression. They provide evidence that Hspb1 expression is regulated by Wnt signalling and that an inhibitor of HSP25 has tumour preventative functions in a mouse model of CRC. Overall, this is an interesting study, which is well presented and concise. The data are mostly clear and the findings are of sufficient interest for publication in EMBO Mol Medicine. Some previous reports indicate HSP27 (the human orthologue) is important for CRC progression but the focus on tumour initiation and prevention here is novel. However, some areas of the study would benefit from further development prior to acceptance:
1) The authors indicate HSP25 inhibition leads to reduced proliferation in Apc deficient cells (Fig 3b) but this doesn't seem to correlate with the findings in Fig 3i-l. If proliferation is transiently inhibited by BVDU treatment in the Lgr5 model then why is tumour number reduced? Could apoptosis of Apc deficient cells also be playing a role? The authors should investigate this in their mouse models. Specifically, is apoptosis induced in the VilCre model at day 3 by BVDU treatment, and is there evidence of apoptosis in the Lgr5 model following BVDU treatment during the critical, early stages of tumour initiation?
2) The authors suggest in their discussion that HSP25 inhibition could be used in patients with FAP. Demonstrating efficacy of BVDU in organoids derived from FAP patients would strengthen the case for this.
3) There is little mechanistic insight into how why HSP25 deletion / inhibition prevents Apc KO growth. The authors could carry out RNAseq and bioinformatics analysis on their Apc vs Apc Hspb1 KO organoids to provide some initial information on potential mechanisms.

Rebuttal document – van Neerven et al. EMM-2022-16194

We thank the editor and reviewers for their thoughtful comments on our manuscript. We very much appreciated the suggestions and recommendations that helped us to significantly improve the study. With the adaptations and substantial amount of novel data and analyses we now provide, we have addressed all points and hope that you will consider the manuscript suitable for publication in *EMBO Molecular Medicine*.

In particular, we have made the following three key adaptations:

1. We provided further functional evidence for effective HSP25/27 inhibition by BVDU *in vitro* and *in vivo*.
2. We have further characterized the stress response following *Apc*-driven oncogenic transformation and provided additional data to strengthen that HSP25 helps channelling this stress.
3. We expanded our findings to relevant human model systems in both organoid cultures derived from familial adenomatous polyposis (FAP) patients and in colorectal cancer cell lines.

Please find below our point-by-point reply. Please also note that we have included the unedited text of the reviewers, but in places we have split and renumbered the queries for clarity, as well as highlighted some phrases.

Referees comments

Referee #1 (Remarks to the Author):
The study by S.M. Van Neerven et al. aims to uncover the role of the protein chaperone HSP25 (HSP27 in humans) during Colorectal Cancer initiation. They used organoids and mouse genetics models of tumor initiation based on APC loss. The authors showed that HSP25 expression is induced in response to Wnt-β-catenin activation and facilitates adenoma transformation. This study describes a novel cancer vulnerability during initiation and therefore is of interest. However, there are important issues that must be addressed:

We thank the reviewer for the accurate summary of our work and the relevant suggestions that have helped us to improve the study considerably.

Major comments:

1.1 My main concern is the use of BVDU as a chemical inhibitor of HSP25: Authors must show functional inhibition of HSP25 by BVDU in their models. BVDU is a thymidine analog with viral specificity, but some of its metabolites inhibit endogenous cellular enzymes (and, for example, synergize with chemotherapy). As most experiments of in-vivo adenoma formation rely on chemical inhibition of HSP25 by BVDU, authors must provide evidence that observed therapeutic effects are due to inhibition of HSP25 activity.

We appreciate this important comment. The role of BVDU as a chemical inhibitor of HSP25/27 has been thoroughly investigated in a previous study that demonstrates BVDU physically binds HSP25/27 and subsequently inhibits its binding to effector proteins (Heinrich *et al*, 2011). We have now elaborated on these findings by incubating HCT116 cancer cells, which are high in HSP27 expression, in the presence of BVDU and collected protein samples over time (**Figure R1**). In doing so, we adapted sample preparation strategy (unboiled in 1% Triton-X100 buffer supplemented with SDS sample buffer) to allow the assessment of HSP polymerization status. It has been reported that HSP25/HSP27 proteins display a reversible structural organization, which is suggested to act as a sensor that

enables adaptation to cellular stress (Katsogiannou *et al*, 2014), with dimers displaying the most active chaperone activity (Hayes *et al*, 2009). We observe that BVDU treatment induces a decrease in HSP27 protein over time, but more specifically we demonstrate loss of the active HSP27 dimers, indicating that BVDU indeed interferes with HSP27 chaperone activity.

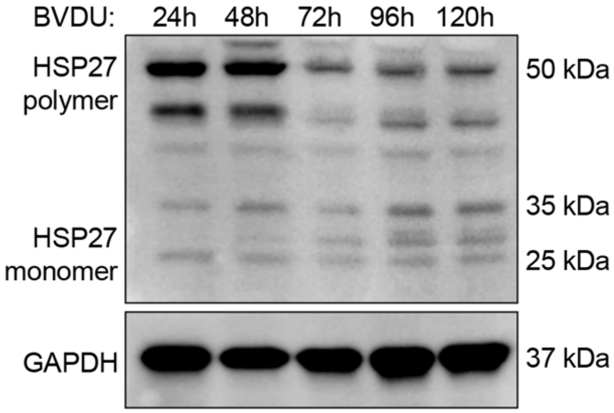

Figure R1. Western blot for HSP27 polymerization in HCT116 cells treated with BVDU.

Moreover, since one of the best studied functions of HSP25/27 is the protection against elevated temperatures, when the intracellular concentration of the 27-kDa heat shock protein increases several-folds, providing thermotolerance by facilitating the recovery phase (Landry *et al*, 1989). To show that BVDU affect thermotolerance, we have now assessed the capacity of our wild type intestinal organoids to recover from heat shock in the presence and absence of BVDU (**Figure R2**). First, we demonstrate that a 60-minute heat shock at 42.5°C results in rapid upregulation of *Hspb1* expression (**Figure R2a**), and concomitant increase in HSP25 protein (**Figure R2b**). Next, we observed that BVDU-treatment reduced the regenerative capacity of intestinal organoids after heat shock (**Figure R2c-e**), indicating that BVDU indeed interferes with recovery from heat shock. We have now added these data to our manuscript.

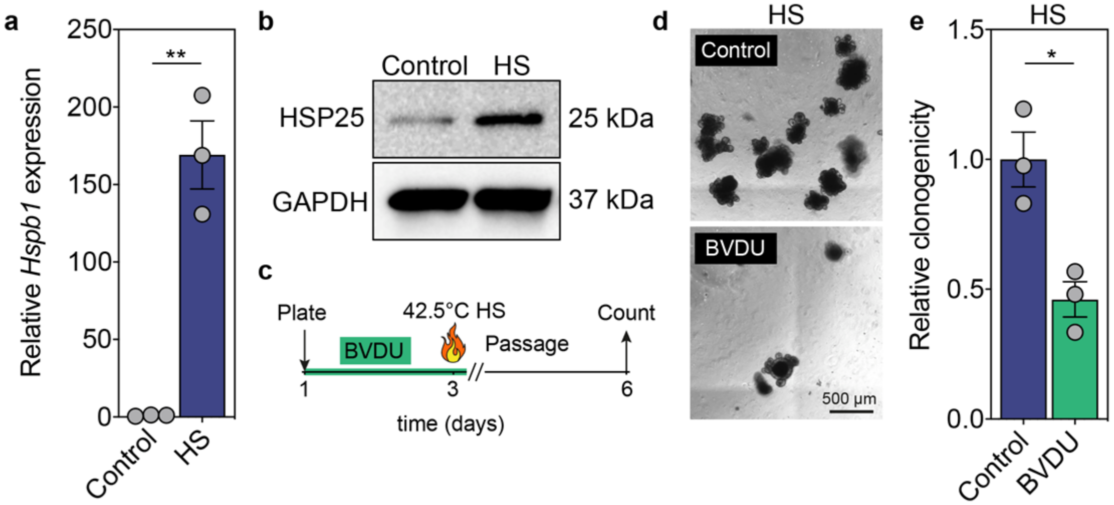

Figure R2. BVDU impairs recovery after heat shock. a, b, Relative *Hspb1* (**a**) and HSP25 (**b**) expression after 1-hour heat shock treatment for 1 hour. **c**, Schematic illustration of experimental strategy for the heat shock recovery assay. **d**, **e**, Representative phase images (**d**) and quantification of clonogenicity (**e**) of wild type organoids passaged after heat shock treatment in the absence or presence of BVDU.

In addition, to strengthen the notion that HSP25 dependency is Wnt regulated, we treated wild type organoids with GSK3-beta inhibitor CHIR99021 in the absence and presence of BVDU. We have previously demonstrated that CHIR99021 treatment results in upregulation of *Hspb1* and HSP25 expression (**Figure R3a, b**), and now reveal that BVDU inhibition also prevents the clonogenic outgrowth of wild type organoids treated with CHIR and BVDU (**Figure R3c, d**).

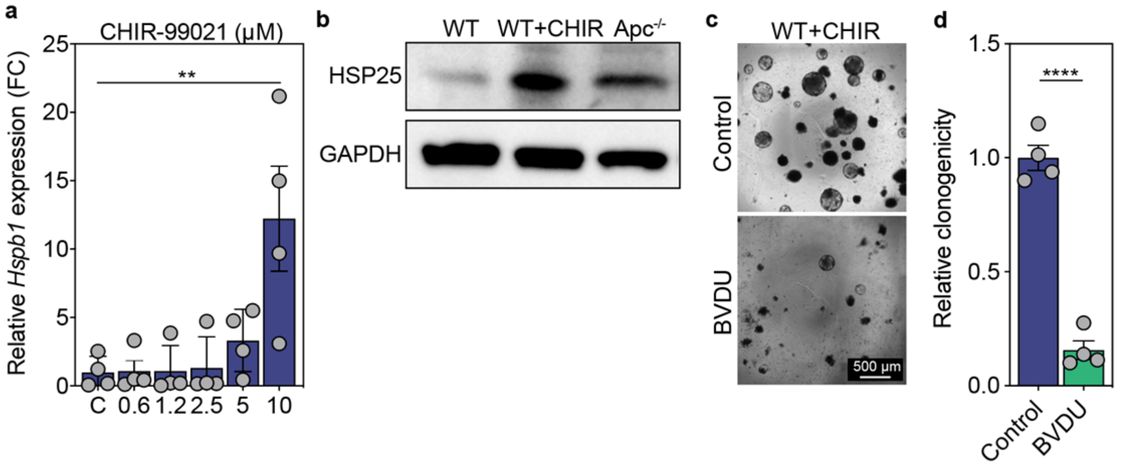

Figure R3. HSP25 dependency is Wnt regulated. a, b, Relative gene expression of *Hspb1* in murine WT organoids treated with different concentrations of CHIR-99021(**a**), and protein expression of HSP25 in WT and *Apc*$^{-/-}$ organoids (**b**). c, d, Representative images (**c**) and clonogenicity (**d**) of WT organoids treated with CHIR99021 in the absence or presence of BVDU.

Moreover, to demonstrate that BVDU prevents *Apc*-driven oncogenic transformation *in vivo,* we have isolated crypts from control or BVDU-treated *Villin-Cre*^{ERT2};*Apc*$^{fl/fl}$ mice 3 days after i.p. tamoxifen injection (**Figure R4a**). Firstly, we have isolated DNA from these crypt fractions and used digital droplet PCR to quantitatively determine the number of unrecombined (*Apc*$^{fl/fl}$) versus recombined (*Apc*$^{-/-}$) *Apc* alleles. We observed that crypts from BVDU-treated mice displayed less recombined (*Apc*$^{-/-}$) alleles than crypts from untreated mice (**Figure R4b**), indicating that fewer *Apc*-mutant cells are present in these fractions. In line with these findings, we also plated equal numbers of crypts in the presence of either ENR (EGF, Noggin, R-spondin) or EN (EGF, Noggin) medium. By using EN medium we specifically select for *Apc*-mutant cells, as wild type cells do not survive in the absence of R-spondin. This experiment reveals that crypts from BVDU treated mice grow out less efficient in EN medium compared to crypts from control mice (**Figure R4c**), indicating that BVDU prevents oncogenic transformation. We have now added these data to our manuscript.

Finally, as discussed in relation to point #2.1 Reviewer 2, treatment of BVDU also induces loss of clonogenic capacity in human CRC lines with high HSP27 expression, which further supports the role of BVDU as a HSP25/27 inhibitor (**Figure R11**).

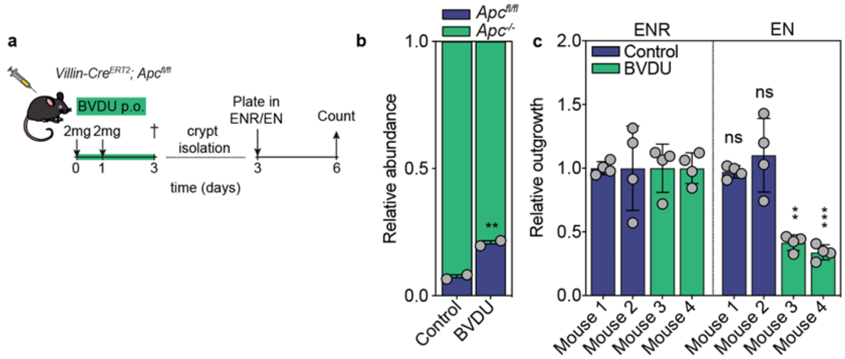

Figure R4. a, Schematic illustration of experimental strategy. **b**, Relative unrecombined (*Apc*$^{fl/fl}$) versus recombined (*Apc*$^{-/-}$) DNA in isolated crypt fractions as assessed by ddPCR. **c**, Clonogenic capacity of isolated crypts in ENR and EN medium.

1.2 A better characterization of Hspb1 KO clones is required. For example, the authors did not demonstrate a loss of HSP25 expression in the Hspb1 KO clones. It is also important to verify whether the effects of BVDU on oncogenic transformation are rescued in Hspb1 KO clones.

This is a valid comment. Naturally, we have validated the knockout of *Hspb1* in our single cell clones. At the DNA level, we reveal that our clones either display a deletion in either exon 1 (KO#1) or exon 2 (KO#2 and #3) (**Figure R5a-c**). Given that our wild type (*Apc*$^{fl/fl}$) organoids hardly express HSP25, and we quickly lose our organoid cultures as soon as we induce oncogenic transformation (*Apc*$^{-/-}$), it is difficult the address loss of HSP25 protein expression in our organoid cultures. However, using a similar approach as described at point #1.1 of this reviewer (**Figure R5d**), we have now demonstrated that our *Hspb1* KO clones do not restore as efficiently as wild type clones after heat shock treatment (**Figure R5e**), indicating that they have an impaired heat shock response, comparable to HSP25 inhibition using BVDU. We have now added these data to our manuscript as **Extended View Figure 2**.

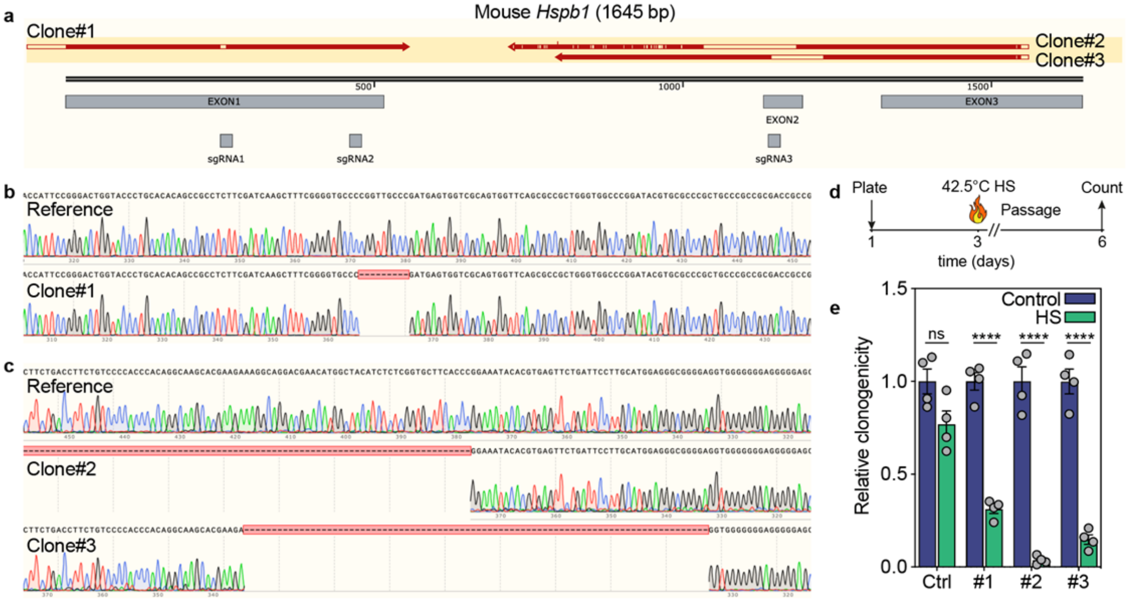

Figure R5. validation of *Hspb1* KO clones. a, Overview of the *Hspb1* gene, and the location of the sgRNA's. **b, c**, Sanger sequencing of three distinct *Hspb1* KO clones at exon 1 (**b**) or exon 2 (**c**). **d**, **e**, functional validation of *Hspb1* KO clones by a heat shock recovery assay (**d**) and the quantification of the clonogenicity after heat shock (**e**).

1.3 The possibility that Apc-mutant cells may depend on HSP25 to cope with proteotoxic stress is appealing. However, induction of proteotoxic stress should be addressed in the models used by the authors, as HSP25 also interacts with other clients involved in apoptosis or cytoskeleton remodeling.

We thank the reviewer for this important point. We and others have previously demonstrated that loss of *Apc* in this organoid system is known to immediately activate a strong Wnt and mTOR-dependent response that leads to strong upregulation of the cell's protein synthesis capacity (Faller *et al*, 2014; Smit *et al*, 2020). The major nascent protein load is considered to elicit some degree of proteotoxic stress, including endoplasmic reticulum (ER)-stress, which explains the marked sensitivity of *Apc*-mutant cells to depletion of chaperones (van Lidth de Jeude *et al*, 2018). We now confirm that, after tamoxifen-mediated recombination, loss of *Apc* readily leads to UPR activation, as shown by increased splicing of *Xbp1* and *Grp78* expression (**Figure R6a, b**). In addition, we found significant enrichment for the HSF1-mediated cellular stress response signature (**Figure R6c**) and chaperone-mediated protein folding signature (**Figure R6d**) in *Apc*-mutant cells, both indicators of proteotoxic stress. Moreover, we also detected an enrichment in the p38/MAPK stress pathway (**Figure R6e**), which is known to be an upstream activator of HSP27 phosphorylation in conditions of stress (Zheng et al. JBC 2006).

Together, this data indicates that *Apc*-driven oncogenic transformation induces an acute proteotoxic stress response and that HSP25 plays a key role in coping with this stress. However, given the many functions of this heat shock protein, we do not exclude the possibility that it is also involved in regulation of e.g. cytoskeletal remodelling and other forms of cellular stress involved in oncogenic transformation. We have now included this data and a discussion on this matter in the revised version of our manuscript.

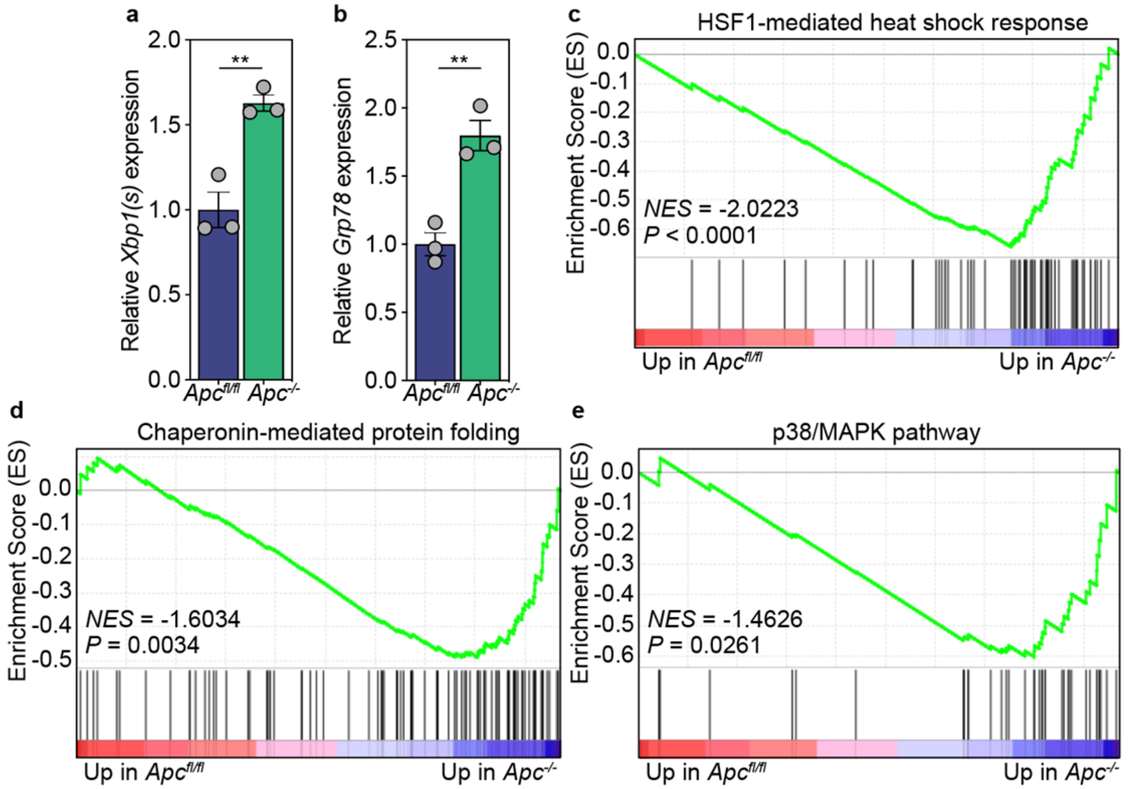

Figure R6. *Apc*-driven oncogenic transformation induces proteotoxic stress. a-e, Loss of *Apc* results in increased splicing of *Xbp1* (**a**), upregulation of *Grp78* expression (**b**), and enrichment for pathways indicative of proteotoxic stress (**c-e**).

1.4 Fig 3b: the authors must show HSP25 protein expression in the epithelium in the presence and absence of 4-OHT administration.

We acknowledge the importance of this point, and to demonstrate that the tamoxifen injection itself does not induce *Hspb1* expression we have now isolated crypt fractions from control and tamoxifen-treated mice 3 days after intraperitoneal injection (**Figure R7a**). This experiment indeed reveals that *Hspb1* expression remains unaffected by tamoxifen administration (**Figure R7b**). Importantly, similar results were obtained when incubating wild type organoids in the absence or presence of tamoxifen *in vitro* (**Figure R7c**). These results have now been added to the manuscript.

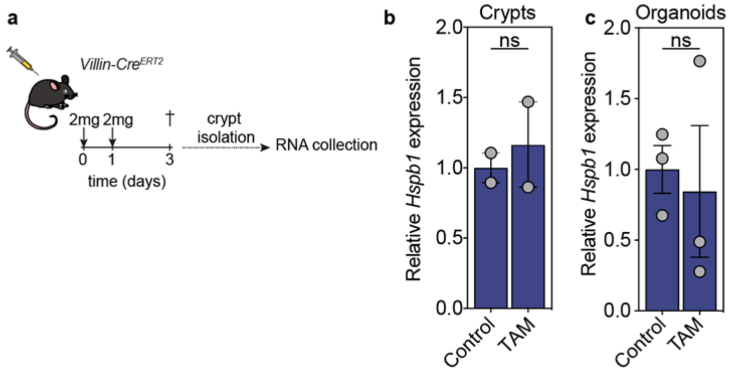

Figure R7. a, Schematic illustration of experimental strategy. **b**, Relative *Hspb1* expression in crypts from control or tamoxifen treated mice. **c**, Relative *Hspb1* expression in control or tamoxifen treated organoids.

1.5 The authors observed a reduction of the Wnt-target genes LGR5 and Axin2 upon BVDU treatment. Do they recapitulate those findings in Hspb1 KO clones? Is it due to stem cell negative selection or to a role of HSP25 in regulating Wnt activity?

This is an interesting point. We have attempted to address this question by collecting RNA the passage after tamoxifen administration using the same experimental setup as our microarray experiment, when the cells develop their cystic-like phenotype. However, as the *Apc*-mutant population is so disadvantaged in our KO clones, we experienced difficulties collecting RNA of sufficient quality. For this reason, we could also not send them for RNA sequencing (as requested by Reviewer 3). We did assess the expression of Wnt target genes *Axin2* and *Lgr5* in the unrecombined (*Apc*$^{fl/fl}$) KO clones and found no differences compared to the control (**Figure R8a, b**). We hope that all additional experiments presented in this rebuttal, in particular the findings presented in **Figure R3**, where we show that elevated Wnt signalling in WT organoids sensitizes them to BVDU treatment, convinces the reviewer that HSP25 has a role in regulating elevated Wnt activation.

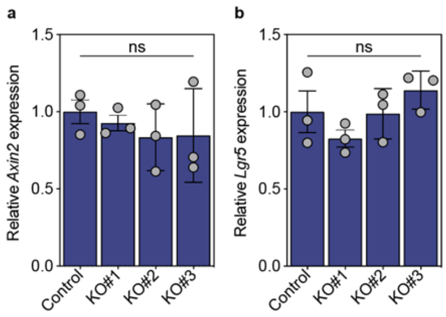

Figure R8. a, b, Relative *Axin2* (**a**) and *Lgr5* (**b**) expression in unrecombined control or CRISPR KO clones.

Minor Comments:

1.6 The authors state: "Together, these data indicate that loss of Apc and the subsequent activation of Wnt signaling directly drives the upregulation of Hspb1 in a TCF-dependent fashion". But, HSP25 is not necessarily a direct target of TCF/LEF, as MYC could also drive its expression. Please discuss this possibility.

We thank the reviewer for pointing this out, we have now adapted the wording in our manuscript.

1.7 Clone size quantification relies exclusively on Notum expression. It is advisable to confirm these results with another adenoma marker, as Notum might be a direct or indirect client of HSP25.

We appreciate this comment and are definitely interested in possible interactions between HSP25 and Wnt pathway proteins. However, we would like to stress that we quantify the clone sizes based on *Notum* mRNA expression, not protein expression. To ensure that BVDU does not influence *Notum* expression levels, we have now checked the effect of BVDU on the expression of *Notum* in our *Apc*-mutant organoids and observed no difference in *Notum* expression between control and treated organoids (**Figure R9**). These findings have been added to the revised manuscript.

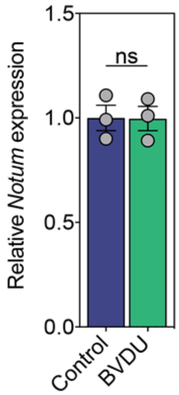

Figure R9. BVDU treatment does not influence expression of Wnt antagonist *Notum*.

1.8 Can the authors explain how they measured "clone fixation"? It seems that these analyses were performed in a steady-state situation.

We apologize for the confusion. We quantify the clone size distributions of intestinal crypts that express *Notum* as proportions of the crypt circumference (from 1:8 to 8:8), clone fixation is subsequently determined as the relative number of crypts that are fully populated by *Notum*+ cells (8:8). Although the measurements for an individual mouse are indeed steady state the progressive increase in fixed clones in time allows to infer dynamical properties of clone expansion as we have employed previously as well (Vermeulen *et al*, 2013; van Neerven *et al*, 2021). In this manuscript, we assessed clone size distributions only at day 14 as, based on our experience with *Apc*-driven dynamics, we anticipate that a decrease in clone fixation at day 14 is sufficient to reduce adenoma formation. We have now adapted the wording in the results section and have elaborated on the quantification strategy in the method section.

1.9 HSP27 has been associated with poor prognosis in various cancers. Can the authors discuss the potential role of HSP25 in established adenocarcinomas?

This is an interesting point, and the reviewer is right that HSP25/27 expression has been associated with a poor prognosis in many cancers. As this was also a request of Reviewer 2,

we have now further characterized the role of *HSPB1* in human CRC cancers in relation to tumour type, tumour stage, and survival (please see points #2.1 and #2.2 of Reviewer 2) and we can confirm that high *HSPB1* expression is indeed associated with reduced survival of CRC patients.

1.10 Please detail how organoid expansion was measured in Supplementary Figure 1 and provide data on BVDU-treated VillinCreERT2;Apc-/- organoids.

We apologize for not being clearer on this important topic. Quantification of organoid growth was done by bright-field microscopic assessment of individual organoids that were cultured in the presence of absence of BVDU. At each time-point, from day 1 to day 4 after seeding, images were taken and the mean organoid area of a minimum of 150 organoids per time-point was quantified using ImageJ software. In quantifications presented in the figures, each biological replicate consists of the mean quantified growth of three wells. We have now included the growth curves for *Apc*-mutant organoids (**Figure R10**) in the manuscript and have added the details of our quantification strategy to the Methods section.

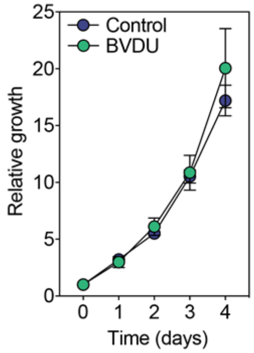

Figure R10. Growth curves of Apc-mutant organoids in the absence or presence of BVDU.

Referee #2 (Comments on Novelty/Model System for Author):

This is a highly relevant and novel study that uses state of the art in vitro (organdies) and in vivo (genetically engineered mice) models and techniques (genetic as well a pharmacological perturbation of intestinal adenomas/organoids). The data as presented are novel, sound and exciting, because they define a novel selective druggable target in APC mutant intestinal adenomas. This is of high clinical relevance for persons at risk for intestinal cancer formation, e.g. due to hereditary syndromes, such as APC mutant familial adenomatous polyposis (FAP).

We wish to thank this reviewer for the favourable assessment of our work and the helpful suggestions that we have all addressed below.

Referee #2 (Remarks to the Author):

In this study, van Neerven and colleagues investigated Wnt driven transcriptional programs during early intestinal cancer formation. During this process, they discovered strong induction of Hspb1, a heat shock protein, which is involved in protein refolding upon cellular stress, apoptosis inhibition, and cytoskeleton remodeling, processes known to be critically regulated by Wnt signalling. This suggests that Hspb1 is a critical executer of Wnt-driven cancer formation. In subsequent experiments, the authors validated this critical function in vitro in organoids and in vivo in mice genetically as well as via pharmacological inhibition. Finally, they show that blockade of Hspb1 specifically decreased the outgrowth Apc tumor cells, but not normal intestinal epithelial cells. This resulted in increased survival of Apc mutant mice.

This is an important study revealing a critical role of the chaperone Hsbp1 for intestinal cancer formation. The techniques used are state of the art and the manuscript is clearly written. The conclusions are supported by the data as presented. However, a few more data and experiments could significantly strengthen this manuscript.

Again, we value these encouraging comments and have taken the suggestion of the expert reviewer to further strengthen our study.

Major concerns:

2.1 Mechanisms of Hsbp1 overexpression and its role in human colorectal cancer: The authors demonstrate convincingly that Hsbp1 is transcriptionally regulated by Wnt pathway activation. Is this the only route of Hsbp1 activation in intestinal cancer? This can be tested e.g. by mining public datasets of human adenomas/CRC for copy number variation/amplification of the Hsbp1 locus or hotspot mutations as well as the epigenetic regulation of Hsbp1.

This is an interesting point. Although we are convinced that in our model systems of tumour initiation *Hspb1* is directly transcriptionally regulated given the marked and very rapid upregulation following *Apc*-loss this does not imply that it is the only relevant mode of regulation.

To address this point we have assessed *HSPB1* expression in relation to the consensus molecular subtypes (CMS, (Guinney *et al*, 2015)) of CRC using the TCGA dataset and the original CMS dataset by Guinney *et al*. These analyses revealed that *HSPB1* expression is significantly increased in tumours with CMS4 subtype (**Figure R11a**). This subtype is enriched in genes implicated in epithelial-to-mesenchymal transition, angiogenesis, matrix remodelling and is associated with poor prognosis (Guinney *et al*, 2015). In addition, cancer from the CMS1 subtype, the microsatellite instable subtype, express significantly more *HSPB1* compared to CMS2 and CMS3 in the dataset from Guinney et al. (**Figure R11a,** right panel) In line with this, we have assessed the relationship between *HSPB1* mRNA expression and HSP27 protein expression in several CRC cell lines that were assigned a CMS subtype and reveal enhanced expression in HCT116 (CMS1) and MDST8 (CMS4) (**Figure R11b, c**). Interestingly, these two lines were more sensitive to BVDU treatment than the others, as assessed using a clonogenic assay (**Figure R11d**), providing further evidence that the BVDU effects are HSP25/27 mediated.

Furthermore, we investigated the methylation status of the *HSPB1* locus at 20 different locations along the locus (**Figure R12a**) and found that *HSPB1* is not methylated in two publicly available datasets of CRC samples (Díez-Villanueva *et al*, 2021; van den Berg *et al*, 2021) and the TCGA database, regardless of tumour location, tumour stage, CMS classification and differentiation grade (**Figure R12b-d**).

Finally, we investigated whether the *HSPB1* locus is frequently gained/lost in CRC using the ASCAT pipeline https://docs.gdc.cancer.gov/Data/Bioinformatics_Pipelines/CNV_Pipeline/. This revealed that almost ~60% of all CRC samples showed a gain of the *HSPB1* locus (262 of the 443 samples, **Figure R13a**), whilst losses were hardly detected (2 of the 443 samples, data not shown). Critically, gains in *HSPB1* are associated with worse overall survival (**Figure R13b**).

Together, this data suggests that *HSPB1* is also of importance in established CRCs, in particular late-stage CRCs, and that the use of BVDU as an anti-cancer treatment might be more widely applicable.

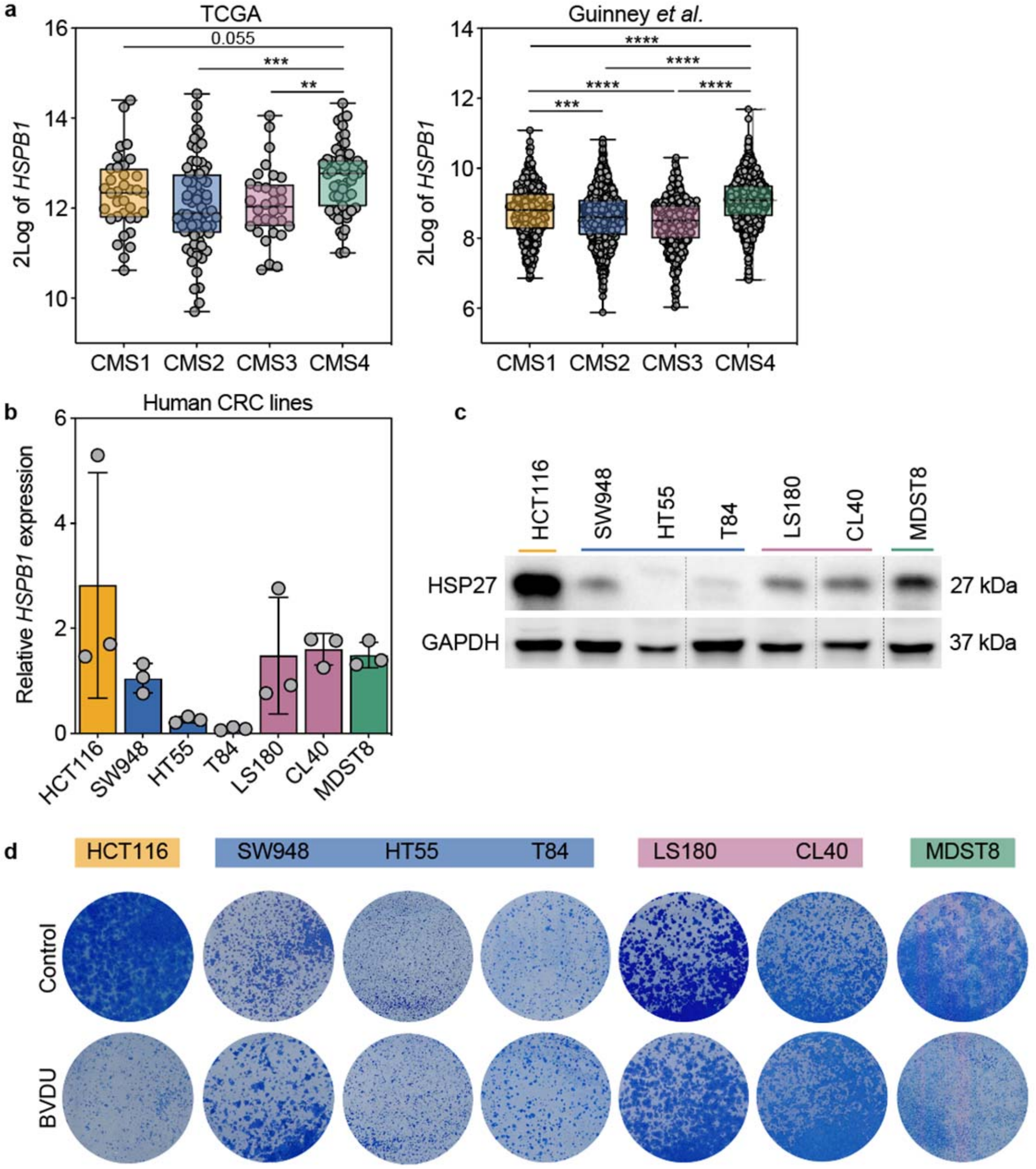

Figure R11. Characterization of *HSPB1* in established CRC. a, Expression of *HSPB1* in the CMS subtypes of CRC using two distinct CRC datasets. **b, c**, *HSPB1* mRNA (**b**) and protein (**c**) expression in CRC cell lines among different CMS subtypes. **d**, Clonogenic assay of CRC cell lines incubated in the absence or presence of BVDU.

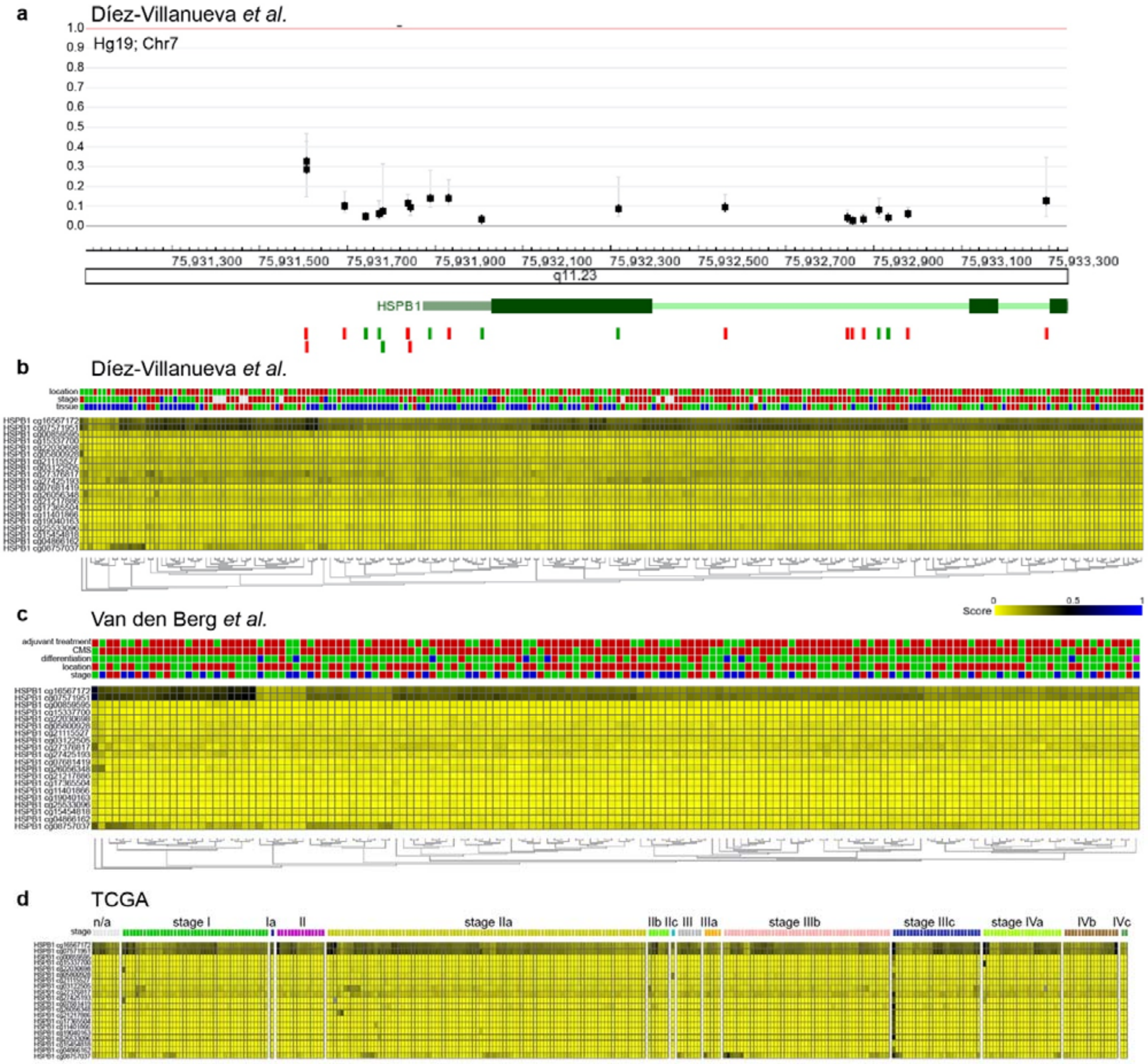

Figure R12. Methylation status of CRC tumours. a, Genome browser view of the location of the 20 probes to assess the methylation status of the *HSPB1* locus using the dataset by Díez-Villanueva. **b-d**, Heatmaps for methylation status measured at 20 locations along the *HSPB1* locus using three distinct datasets. **b**, Dataset by Díez-Villanueva *et al.* containing normal mucosa or stage IIa CRCs. Sample location (red: left colon, green: right colon), tumour stage (red: n/a, green: stage IIa), tissue type (red: mucosa healthy donor, green: mucosa CRC patient, blue: tumour tissue). **c,** Dataset by van der Berg *et al*. containing CMS2 and CMS3 samples. Adjuvant treatment (red: no, green: yes), CMS classification (red: CMS2, green: CMS3), Differentiation status (red: good, green: moderate, blue: poor, purple: unknown), tumour location (red: left, green: right), tumour stage (red: stage I, green: stage II, blue: stage III). **d**, TCGA dataset of CRC samples, separated by tumour stage.

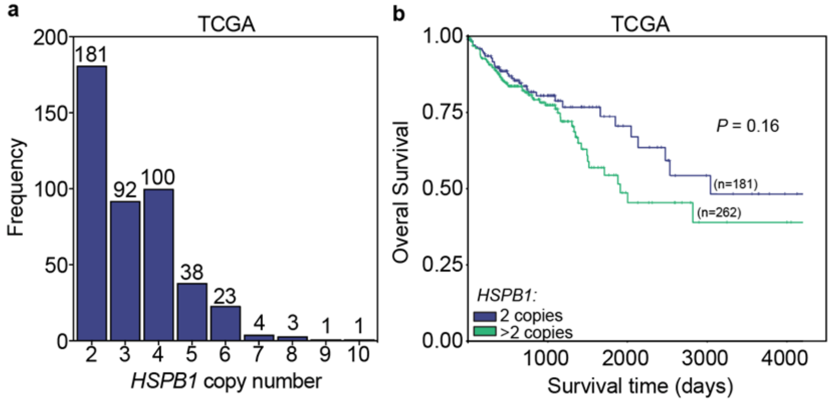

Figure R13. ***HSPB1* Copy number analysis. a**, Distribution of *HSPB1* copy numbers within the TCGA dataset. **b**, Overall survival of patients without (2 copies) or with (>2 copies) copy number gains in *HSPB1*.

2.2 Is Hsbp1 overexpression associated with a poor patient survival or other important clinical features, such as tumor differentiation and metastasis? Does BVDU treatment induces morphological changes or apoptosis of the adenomas?

As demonstrated at point #2.1, *HSPB1* expression seems to be highest in the poor prognosis CMS4 subtype, it's locus is generally not methylated and copy number gains are often observed among CRC patients. We have now evaluated the association of *HSPB1* with tumour stage in two distinct CRC datasets and reveal that *HSPB1* expression is significantly increased in the later tumour stages (Stage 3-4) (**Figure R14a, b**). In line with these findings, high *HSPB1* expression is also associated with a significant decrease in overall survival.

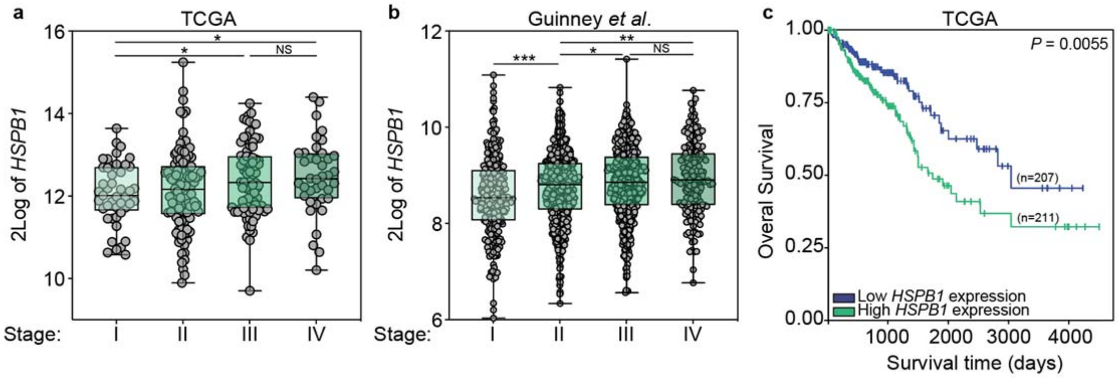

Figure R14. a, b, *HSPB1* expression in two distinct datasets, separated by tumour stage. **c**, Overall survival of patients with low or high expression levels of *HSPB1*.

Moreover, both control and BVDU-treated adenomas displayed their typical glandular structure, and we did not detect any differences in apoptosis levels as assessed using cleaved caspase 3 staining (**Figure R15**). However, we would like to stress that we only treated the mice in the first 10 days after tamoxifen injection to prevent the establishment and expansion of *Apc*-mutant cells. The resulting adenomas that grew out after this stage have therefore not been exposed to BVDU treatment.

Given that our manuscript focuses on prevention of tumour initiation, we have currently not included any data regarding the relationship between *HSPB1* and later-stage CRC in our manuscript. Naturally, we are willing to include these data in our manuscript of this is the wish of the editor and/or the reviewers.

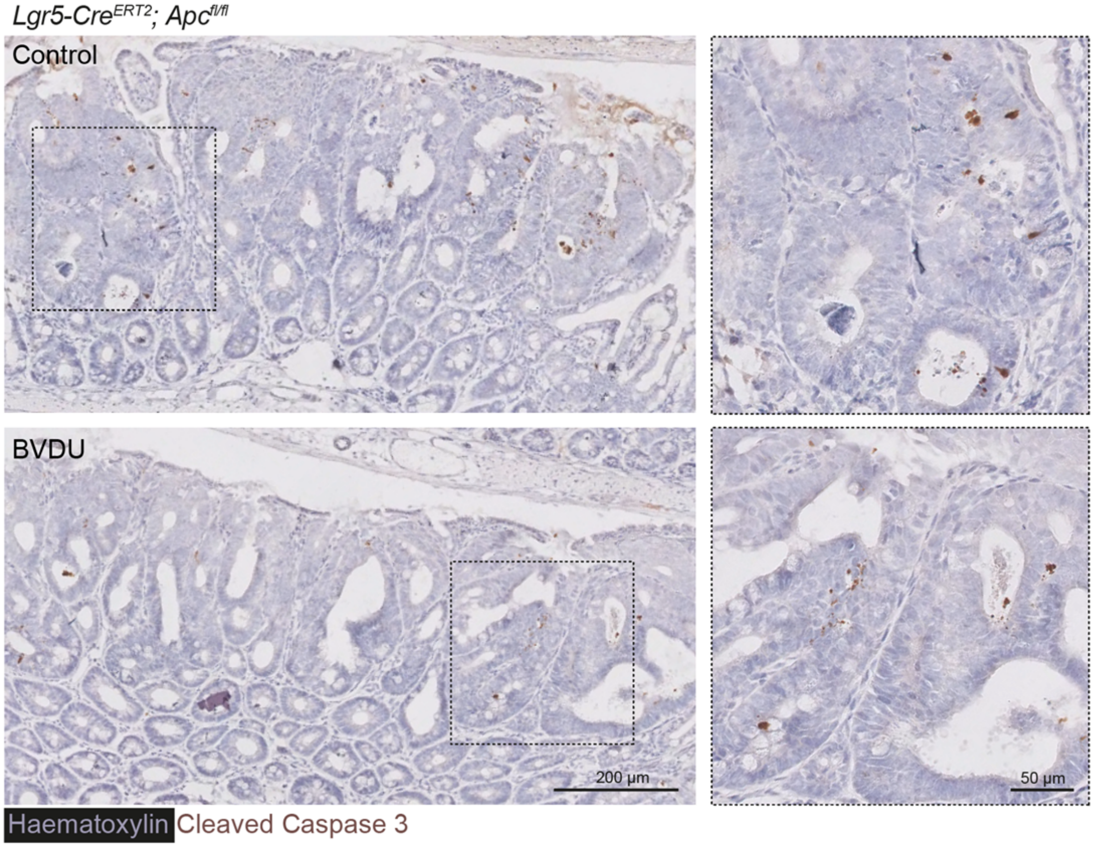

Figure R15. Morphology and apoptosis levels in adenomas from control and BVDU treated mice.

2.3 What´s the mode of action of BVDU? More data on potential side effects (e.g. weight loss or other adverse effects of treated mice) and off target effects of BVDU would strengthen the translational impact of the manuscript.

We thank the reviewer for this suggestion. The proposed treatment regime (400 mg/kg daily) was well-tolerated by our mice and did not result in noticeable side-effects nor did it influence the weight of the mice as demonstrated in **Figure R16**. Moreover, a similar BVDU dose and treatment regimen has been used in a previously published study (De Clercq *et al*, 1982). More importantly, there are several clinical studies on the effects of BVDU for the treatment of herpes zoster, herpetic keratitis, and VZV infections, and these studies report minimal side effects in adults or children (Wassilew, 2005; Wildiers & De Clercq, 1984; Benoit *et al*, 1985), with the most common side effect observed among several clinical trials being nausea in ~2% of all patients (Salvaggio & Gnann, 2017). We have now included a discussion on these findings in our manuscript.

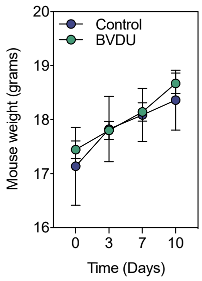

Figure R16. Weight of the BVDU-treated mice during the 10-day treatment regime, compared to control mice.

Referee #3 (Comments on Novelty/Model System for Author):

The models used are appropriate and the technical quality is good. There is high potential for medical impact. The novelty is high for the model system being used but similar results have been reported in other systems. This doesn't detract from the overall potential impact of the work.

We appreciate the comments by this reviewer and agree with the potential clinical impact of this work in the future.

Referee #3 (Remarks for Author):

In this report Neerven and colleagues identify a dependency for Apc mutant cells on the heat shock protein HSP25. They outline a number of experiments detailing a transcriptional upregulation of Hspb1 (which encodes HSP25) following Apc loss and the requirement of Apc KO cells on its expression. They provide evidence that Hspb1 expression is regulated by Wnt signalling and that an inhibitor of HSP25 has tumour preventative functions in a mouse model of CRC. Overall, this is an interesting study, which is well presented and concise. The data are mostly clear and the findings are of sufficient interest for publication in EMBO Mol Medicine. Some previous reports indicate HSP27 (the human orthologue) is important for CRC progression but the focus on tumour initiation and prevention here is novel. However, some areas of the study would benefit from further development prior to acceptance:

We thank the reviewer for this positive assessment of our study and have used the comments to further strengthen the manuscript.

3.1 The authors indicate HSP25 inhibition leads to reduced proliferation in Apc deficient cells (Fig 3b) but this doesn't seem to correlate with the findings in Fig 3i-l. If proliferation is transiently inhibited by BVDU treatment in the Lgr5 model then why is tumour number reduced? Could apoptosis of Apc deficient cells also be playing a role? The authors should investigate this in their mouse models. Specifically, is apoptosis induced in the VilCre model at day 3 by BVDU treatment, and is there evidence of apoptosis in the Lgr5 model following BVDU treatment during the critical, early stages of tumour initiation?

We thank the reviewer for raising this important question. What we have attempted to investigate in the experiment underlying Figure 3b is whether the hyperproliferative phenotype as a result of epithelial-wide loss of *Apc* is reduced in mice treated with BVDU. This model has been described previously and reveals how hyperproliferation is a direct result of oncogenic transformation (Sansom *et al*, 2004). Therefore, with this experimental setup, we do not specifically inhibit proliferation, but prevent transformation towards highly proliferative *Apc*-mutant cells. We have now clarified this by demonstrating that crypts isolated from BVDU-treated mice contain fewer recombined *Apc* alleles (*Apc*$^{-/-}$), and that these grow out less efficiently in medium without R-spondin1 (which selects for *Apc*-mutant cells) (**Figure R4, point #1.1**).

3.2 The authors suggest in their discussion that HSP25 inhibition could be used in patients with FAP. Demonstrating efficacy of BVDU in organoids derived from FAP patients would strengthen the case for this.

This is a valid point. We have first confirmed increased expression of *HSPB1* in human organoids derived from FAP (*APC*$^{-/-}$) patients compared to normal colon organoids (*APC*$^{+/+}$) (**Figure R17a**), which shows marked upregulation of *HSPB1* in *APC* mutant organoids. Next, we have treated FAP organoids with BVDU and observed a general reduction in clonogenic capacity, whilst normal organoids were unaffected (**Figure R17b, c**). Together, this data supports the notion that BVDU also inhibits HSP27 and affects clonogenic capacity of

human FAP organoids in a similar fashion as the murine mutants. This data has now been included in the revised manuscript.

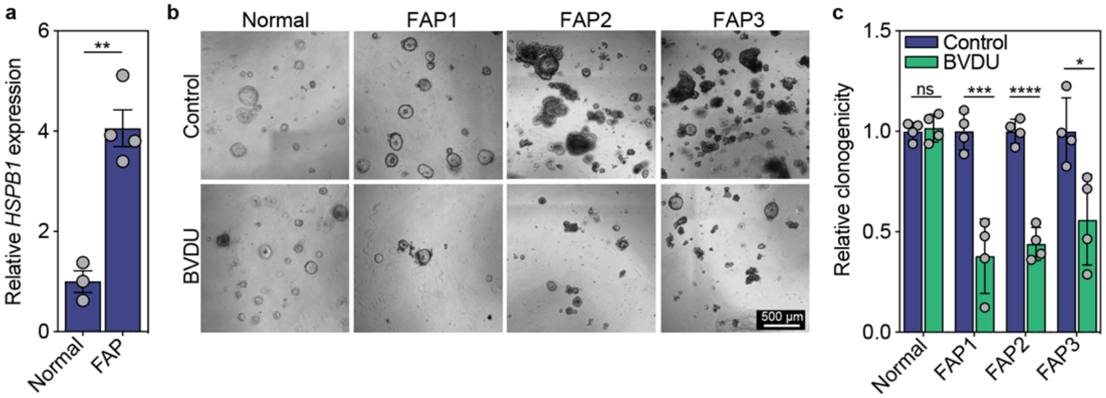

Figure R17. a, Relative *HSPB1* expression in human colon organoids from normal colon or FAP adenomas. **b**, **c**, representative images (**b**) and quantification (**c**) of clonogenic capacity of human colon organoids in the absence or presence of BVDU.

3.3 There is little mechanistic insight into how why HSP25 deletion / inhibition prevents Apc KO growth. The authors could carry out RNAseq and bioinformatics analysis on their Apc vs Apc Hspb1 KO organoids to provide some initial information on potential mechanisms.

We thank the reviewer for this suggestion. We have attempted to address this question, however, due to the disadvantage that *Apc*-mutant organoids experience upon loss of *Hspb1* we are not able to collect mRNA of sufficient quality to perform RNA sequencing. Instead, we have now performed more functional experiments to validate both BVDU and the *Hspb1* KO clones (Point #1.1 and 1.2), included more in-depth transcriptional analysis of the stress response of our *Apc*-mutant organoids (Point #1.3), and provided human data (Point #2.1 and #2.2). With all these new results, we hope to convince the reviewer that that *Apc*-loss indeed induces a stress response and that *Hspb1*/HSP25 helps in channelling this stress.

References

Benoit Y, Laureys G, Delbeke MJ & De Clercq E (1985) Oral BVDU treatment of varicella and zoster in children with cancer. *Eur J Pediatr* 143: 198–202

van den Berg I, Smid M, Coebergh van den Braak RRJ, van de Wiel MA, van Deurzen CHM, de Weerd V, Martens JWM, IJzermans JNM & Wilting SM (2021) A panel of DNA methylation markers for the classification of consensus molecular subtypes 2 and 3 in patients with colorectal cancer. *Mol Oncol* 15: 3348–3362

De Clercq E, Zhang ZX & Sim IS (1982) Treatment of experimental herpes simplex virus encephalitis with (E)-5-(2-bromovinyl)-2'-deoxyuridine in mice. *Antimicrob Agents Chemother* 22: 421–425

Díez-Villanueva A, Jordà M, Carreras-Torres R, Alonso H, Cordero D, Guinó E, Sanjuan X, Santos C, Salazar R, Sanz-Pamplona R, *et al* (2021) Identifying causal models between genetically regulated methylation patterns and gene expression in healthy colon tissue. *Clin Epigenetics* 13

Faller WJ, Jackson TJ, Knight JRP, Ridgway RA, Jamieson T, Karim SA, Jones C, Radulescu S, Huels DJ, Myant KB, *et al* (2014) mTORC1-mediated translational elongation limits intestinal tumour initiation and growth. *Nature* 517: 497–500

Guinney J, Dienstmann R, Wang X, de Reyniès A, Schlicker A, Soneson C, Marisa L, Roepman P, Nyamundanda G, Angelino P, *et al* (2015) The consensus molecular subtypes of colorectal cancer. *Nat Med* 21: 1350–1356

Hayes D, Napoli V, Mazurkie A, Stafford WF & Graceffa P (2009) Phosphorylation dependence of Hsp27 multimeric size and molecular chaperone function. *J Biol Chem*

284: 18801–18807
Van Der Heijden M, Zimberlin CD, Nicholson AM, Colak S, Kemp R, Meijer SL, Medema JP, Greten FR, Jansen M, Winton DJ, *et al* (2016) Bcl-2 is a critical mediator of intestinal transformation. *Nat Commun* 7: 1–11
Heinrich JC, Tuukkanen A, Schroeder M, Fahrig T & Fahrig R (2011) RP101 (Brivudine) binds to heat shock protein HSP27 (HSPB1) and enhances survival in animals and pancreatic cancer patients. *J Cancer Res Clin Oncol* 137: 1349–1361
Katsogiannou M, Andrieu C & Rocchi P (2014) Heat shock protein 27 phosphorylation state is associated with cancer progression. *Front Genet* 5: 346
Landry J, Chretien P, Lambert H, Hickey E & Weber LA (1989) Heat shock resistance conferred by expression of the human HSP27 gene in rodent cells. *J Cell Biol* 109: 7–15
van Lidth de Jeude JF, Spaan CN, Meijer BJ, Smit WL, Soeratram TTD, Wielenga MCB, Westendorp BF, Lee AS, Meisner S, Vermeulen JLM, *et al* (2018) Heterozygosity of Chaperone Grp78 Reduces Intestinal Stem Cell Regeneration Potential and Protects against Adenoma Formation. *Cancer Res* 78: 6098–6106
van Neerven SM, de Groot NE, Nijman LE, Scicluna BP, van Driel MS, Lecca MC, Warmerdam DO, Kakkar V, Moreno LF, Vieira Braga FA, *et al* (2021) Apc-mutant cells act as supercompetitors in intestinal tumour initiation. *Nature* 594: 436–441
Salvaggio MR & Gnann JW (2017) Drugs for Herpesvirus Infections. *Infect Dis (Auckl)*: 1309-1317.e1
Sansom OJ, Reed KR, Hayes AJ, Ireland H, Brinkmann H, Newton IP, Batlle E, Simon-Assmann P, Clevers H, Nathke IS, *et al* (2004) Loss of Apc in vivo immediately perturbs Wnt signaling, differentiation, and migration. *Genes Dev* 18: 1385–1390
Smit WL, Spaan CN, De Boer RJ, Ramesh P, Garcia TM, Meijer BJ, Vermeulen JLM, Lezzerini M, MacInnes AW, Koster J, *et al* (2020) Driver mutations of the adenoma-carcinoma sequence govern the intestinal epithelial global translational capacity. *Proc Natl Acad Sci U S A* 117: 25560–25570
Vermeulen L, Morrissey E, van der Heijden M, Nicholson AM, Sottoriva A, Buczacki S, Kemp R, Tavaré S & Winton DJ (2013) Defining stem cell dynamics in models of intestinal tumor initiation. *Science* 342: 995–8
Wassilew SW (2005) Brivudin compared with famciclovir in the treatment of herpes zoster: effects in acute disease and chronic pain in immunocompetent patients. A randomized, double-blind, multinational study. *J Eur Acad Dermatology Venereol* 19: 47–55
Wildiers J & De Clercq E (1984) Oral (E)-5-(2-bromovinyl)-2’-deoxyuridine treatment of severe herpes zoster in cancer patients. *Eur J Cancer Clin Oncol* 20: 471–476

7th Oct 2022

Dear Prof. Vermeulen,

Thank you for the submission of your manuscript to EMBO Molecular Medicine, and please accept my apologies for the delay in getting back to you as one referee needed more time to review the manuscript. As you will see below, the three referees are now supportive of publication, and I am therefore pleased to inform you that we will be able to accept your manuscript once the following editorial points will be addressed:

1/ Manuscript text:
- Please accept the changes, remove the highlights in the text, and only keep in track changes mode any new modification.
- Material and methods:
o Animals: Please indicate the origin of the mice.
o Human samples: Include a statement that the experiments conformed to the principles set out in the WMA Declaration of Helsinki and the Department of Health and Human Services Belmont Report.
- Thank you for providing the Data Availability section. Kindly only mention: "This study includes no data deposited in external repositories."

2/ Thank you for providing a synopsis text. Please note that this would be the final version and changes during proofing are usually not allowed.
Please also suggest a visual abstract to illustrate your article as a PNG/TIFF/JPEG file 550 px wide x 300-600 px high.

3/ As part of the EMBO Publications transparent editorial process initiative (see our Editorial at http://embomolmed.embopress.org/content/2/9/329), EMBO Molecular Medicine will publish online a Review Process File (RPF) to accompany accepted manuscripts.
This file will be published in conjunction with your paper and will include the anonymous referee reports, your point-by-point response and all pertinent correspondence relating to the manuscript. Let us know whether you agree with the publication of the RPF and as here, if you want to remove or not any figures from it prior to publication.
Please note that the Authors checklist will be published at the end of the RPF.

I look forward to receiving your revised manuscript.

Yours sincerely,

Lise Roth

Lise Roth, PhD
Senior Editor
EMBO Molecular Medicine

***** Reviewer's comments *****

Referee #1 (Remarks for Author):

The authors satisfyingly addressed most of my comments and convincingly demonstrated that BVDU inhibits HSP25/27. The role of HSP25/27 in protecting from proteotoxic stress, especially in Apc mutant cells, could have been further characterized in my opinion. However, this does not impede the publication of this manuscript in EMBO Molecular Medicine in its current form.

Referee #2 (Comments on Novelty/Model System for Author):

This highly relevant study uses state of the art in vitro (organoids) and in vivo (genetically engineered mice) models and techniques.

Referee #2 (Remarks for Author):

The authors have addressed all my suggestions and concerns. The revised manuscript ready very well, the data are sound, novel and of high clinical relevance. I have no additional comments or concerns that should be addressed.

Referee #3 (Remarks for Author):

The authors have suitably addressed my comments and I now recommend publication.

The authors performed the requested editorial changes.

12th Oct 2022

Dear Prof. Vermeulen,

Thank you for sending the revised files. I am pleased to inform you that your manuscript is accepted for publication and is now being sent to our publisher to be included in the next available issue of EMBO Molecular Medicine!

Congratulations on your interesting work,

With my best wishes,

Lise

Lise Roth, Ph.D
Senior Editor
EMBO Molecular Medicine

Follow us on Twitter @EmboMolMed
Sign up for eTOCs at embopress.org/alertsfeeds

EMBO Press Author Checklist

Corresponding Author Name: s.m.vanneerven@amsterdamumc.nl & l.vermeulen@amsterdamumc.nl	
Journal Submitted to: EMBO Molecular Medicine	
Manuscript Number: 2022-16194	

USEFUL LINKS FOR COMPLETING THIS FORM
The EMBO Journal - Author Guidelines
EMBO Reports - Author Guidelines
Molecular Systems Biology - Author Guidelines
EMBO Molecular Medicine - Author Guidelines

Reporting Checklist for Life Science Articles (updated January 2022)
This checklist is adapted from Materials Design Analysis Reporting (MDAR) Checklist for Authors. MDAR establishes a minimum set of requirements in transparent reporting in the life sciences (see Statement of Task: 10.31222/osf.io/9sm4x). Please follow the journal's guidelines in preparing your manuscript.
Please note that a copy of this checklist will be published alongside your article.

Abridged guidelines for figures
1. Data
The data shown in figures should satisfy the following conditions:
- the data were obtained and processed according to the field’s best practice and are presented to reflect the results of the experiments in an accurate and unbiased manner.
- ideally, figure panels should include only measurements that are directly comparable to each other and obtained with the same assay.
- plots include clearly labeled error bars for independent experiments and sample sizes. Unless justified, error bars should not be shown for technical replicates.
- if n<5, the individual data points from each experiment should be plotted. Any statistical test employed should be justified.
- Source Data should be included to report the data underlying figures according to the guidelines set out in the authorship guidelines on Data Presentation.

2. Captions
Each figure caption should contain the following information, for each panel where they are relevant:
- a specification of the experimental system investigated (eg cell line, species name).
- the assay(s) and method(s) used to carry out the reported observations and measurements.
- an explicit mention of the biological and chemical entity(ies) that are being measured.
- an explicit mention of the biological and chemical entity(ies) that are altered/varied/perturbed in a controlled manner.
- the exact sample size (n) for each experimental group/condition, given as a number, not a range;
- a description of the sample collection allowing the reader to understand whether the samples represent technical or biological replicates (including how many animals, litters, cultures, etc.).
- a statement of how many times the experiment shown was independently replicated in the laboratory.
- definitions of statistical methods and measures:
  - common tests, such as t-test (please specify whether paired vs. unpaired), simple χ2 tests, Wilcoxon and Mann-Whitney tests, can be unambiguously identified by name only, but more complex techniques should be described in the methods section;
  - are tests one-sided or two-sided?
  - are there adjustments for multiple comparisons?
  - exact statistical test results, e.g., P values = x but not P values < x;
  - definition of ‘center values’ as median or average;
  - definition of error bars as s.d. or s.e.m.

Please complete ALL of the questions below.
Select "Not Applicable" only when the requested information is not relevant for your study.

Materials

Newly Created Materials	Information included in the manuscript?	In which section is the information available? (Reagents and Tools Table, Materials and Methods, Figures, Data Availability Section)
New materials and reagents need to be available; do any restrictions apply?	Not Applicable	

Antibodies	Information included in the manuscript?	In which section is the information available? (Reagents and Tools Table, Materials and Methods, Figures, Data Availability Section)
For **antibodies** provide the following information:<br>- Commercial antibodies: RRID (if possible) or supplier name, catalogue number and or/clone number<br>- Non-commercial: RRID or citation	Yes	Method section

DNA and RNA sequences	Information included in the manuscript?	In which section is the information available? (Reagents and Tools Table, Materials and Methods, Figures, Data Availability Section)
**Short novel DNA or RNA including primers, probes**: provide the sequences.	Yes	Method section

Cell materials	Information included in the manuscript?	In which section is the information available? (Reagents and Tools Table, Materials and Methods, Figures, Data Availability Section)
**Cell lines:** Provide species information, strain. Provide accession number in repository **OR** supplier name, catalog number, clone number, and/**OR** RRID.	Yes	Method section
**Primary cultures:** Provide species, strain, sex of origin, genetic modification status.	Yes	Method section
Report if the cell lines were recently **authenticated** (e.g., by STR profiling) and tested for mycoplasma contamination.	Yes	Method section

Experimental animals	Information included in the manuscript?	In which section is the information available? (Reagents and Tools Table, Materials and Methods, Figures, Data Availability Section)
**Laboratory animals or Model organisms:** Provide species, strain, sex, age, genetic modification status. Provide accession number in repository **OR** supplier name, catalog number, clone number, **OR** RRID.	Yes	Method section
**Animal observed in or captured from the field:** Provide species, sex, and age where possible.	Not Applicable	
Please detail **housing and husbandry conditions**.	Yes	Method section

Plants and microbes	Information included in the manuscript?	In which section is the information available? (Reagents and Tools Table, Materials and Methods, Figures, Data Availability Section)
**Plants:** provide species and strain, ecotype and cultivar where relevant, unique accession number if available, and source (including location for collected wild specimens).	Not Applicable	
**Microbes:** provide species and strain, unique accession number if available, and source.	Not Applicable	

Human research participants	Information included in the manuscript?	In which section is the information available? (Reagents and Tools Table, Materials and Methods, Figures, Data Availability Section)
If collected and within the bounds of privacy constraints report on age, sex and gender or ethnicity for all study participants.	Yes	Method section

Core facilities	Information included in the manuscript?	In which section is the information available? (Reagents and Tools Table, Materials and Methods, Figures, Data Availability Section)
If your work benefited from core facilities, was their service mentioned in the acknowledgments section?	Not Applicable	

Design

Study protocol	Information included in the manuscript?	In which section is the information available? (Reagents and Tools Table, Materials and Methods, Figures, Data Availability Section)
If study protocol has been **pre-registered, provide DOI in the manuscript**. For clinical trials, provide the trial registration number **OR** cite DOI.	Not Applicable	
Report the **clinical trial registration number** (at ClinicalTrials.gov or equivalent), where applicable.	Not Applicable	

Laboratory protocol	Information included in the manuscript?	In which section is the information available? (Reagents and Tools Table, Materials and Methods, Figures, Data Availability Section)
Provide DOI OR other citation details if **external detailed step-by-step protocols** are available.	Not Applicable	

Experimental study design and statistics	Information included in the manuscript?	In which section is the information available? (Reagents and Tools Table, Materials and Methods, Figures, Data Availability Section)
Include a statement about **sample size** estimate even if no statistical methods were used.	Yes	Method section
Were any steps taken to minimize the effects of subjective bias when allocating animals/samples to treatment (e.g. **randomization procedure**)? If yes, have they been described?	Yes	Method section
Include a statement about **blinding** even if no blinding was done.	Yes	Method section
Describe **inclusion/exclusion criteria** if samples or animals were excluded from the analysis. Were the criteria pre-established?<br><br>If sample or data points were omitted from analysis, report if this was due to attrition or intentional exclusion and provide justification.	Yes	Method section
For every figure, are **statistical tests** justified as appropriate? Do the data meet the assumptions of the tests (e.g., normal distribution)? Describe any methods used to assess it. Is there an estimate of variation within each group of data? Is the variance similar between the groups that are being statistically compared?	Yes	Method section

Sample definition and in-laboratory replication	Information included in the manuscript?	In which section is the information available? (Reagents and Tools Table, Materials and Methods, Figures, Data Availability Section)
In the figure legends: state number of times the experiment was **replicated** in laboratory.	Yes	Figure legends
In the figure legends: define whether data describe **technical or biological replicates**.	Yes	Figure legends

Ethics

Ethics	Information included in the manuscript?	In which section is the information available? (Reagents and Tools Table, Materials and Methods, Figures, Data Availability Section)
Studies involving **human participants**: State details of **authority granting ethics approval** (IRB or equivalent committee(s), provide reference number for approval.	Yes	Method section
Studies involving **human participants**: Include a statement confirming that **informed consent** was obtained from all subjects and that the experiments conformed to the principles set out in the WMA Declaration of Helsinki and the Department of Health and Human Services Belmont Report.	Yes	Method section
Studies involving **human participants:** For publication of **patient photos**, include a statement confirming that consent to publish was obtained.	Not Applicable	
Studies involving experimental **animals**: State details of **authority granting ethics approval** (IRB or equivalent committee(s), provide reference number for approval. Include a statement of compliance with ethical regulations.	Yes	Method section
Studies involving **specimen and field samples:** State if relevant **permits** obtained, provide details of authority approving study; if none were required, explain why.	Not Applicable	

Dual Use Research of Concern (DURC)	Information included in the manuscript?	In which section is the information available? (Reagents and Tools Table, Materials and Methods, Figures, Data Availability Section)
Could your study fall under dual use research restrictions? Please check biosecurity documents and list of **select agents and toxins** (CDC): https://www.selectagents.gov/sat/list.htm	Not Applicable	
If you used a select agent, is the security level of the lab appropriate and reported in the manuscript?	Not Applicable	
If a study is subject to dual use research of concern regulations, is the name of the **authority granting approval and reference number** for the regulatory approval provided in the manuscript?	Not Applicable	

Reporting

The MDAR framework recommends adoption of discipline-specific guidelines, established and endorsed through community initiatives. Journals have their own policy about requiring specific guidelines and recommendations to complement MDAR.

Adherence to community standards	Information included in the manuscript?	In which section is the information available? (Reagents and Tools Table, Materials and Methods, Figures, Data Availability Section)
State if relevant guidelines or checklists (e.g., **ICMJE, MIBBI, ARRIVE, PRISMA**) have been followed or provided.	Not Applicable	
For **tumor marker prognostic studies,** we recommend that you follow the **REMARK** reporting guidelines (see link list at top right). See author guidelines, under 'Reporting Guidelines'. Please confirm you have followed these guidelines.	Not Applicable	
For **phase II and III randomized controlled trials**, please refer to the **CONSORT** flow diagram (see link list at top right) and submit the CONSORT checklist (see link list at top right) with your submission. See author guidelines, under 'Reporting Guidelines'. Please confirm you have submitted this list.	Not Applicable	

Data Availability

Data availability	Information included in the manuscript?	In which section is the information available? (Reagents and Tools Table, Materials and Methods, Figures, Data Availability Section)
Have **primary datasets** been deposited according to the journal's guidelines (see 'Data Deposition' section) and the respective accession numbers provided in the Data Availability Section?	Not Applicable	
Were **human clinical and genomic datasets** deposited in a public access-controlled repository in accordance to ethical obligations to the patients and to the applicable consent agreement?	Not Applicable	
Are **computational models** that are central and integral to a study available without restrictions in a machine-readable form? Were the relevant accession numbers or links provided?	Not Applicable	
If publicly available data were reused, provide the respective **data citations in the reference list**.	Yes	Reference list
